# Advancements and challenges in brain cancer therapeutics

**DOI:** 10.1002/EXP.20230177

**Published:** 2024-05-16

**Authors:** Fan Bai, Yueyang Deng, Long Li, Ming Lv, Jamoliddin Razzokov, Qingnan Xu, Zhen Xu, Zhaowei Chen, Guojun Chen, Zhitong Chen

**Affiliations:** ^1^ Paul C Lauterbur Research Center for Biomedical Imaging, Institute of Biomedical and Health Engineering Shenzhen Institute of Advanced Technology Chinese Academy of Sciences Shenzhen China; ^2^ Advanced Therapeutic Center National Innovation Center for Advanced Medical Devices Shenzhen China; ^3^ Department of Biomedical Engineering McGill University Montreal Quebec Canada; ^4^ Rosalind & Morris Goodman Cancer Institute McGill University Montreal Quebec Canada; ^5^ University of Chinese Academy of Sciences Shenzhen Guangdong P. R. China; ^6^ Department of Medical Engineering Medical Supplies Center of Chinese PLA General Hospital Beijing China; ^7^ Institute of Fundamental and Applied Research National Research University TIIAME Tashkent Uzbekistan; ^8^ Laboratory of Experimental Biophysics Centre for Advanced Technologies Tashkent Uzbekistan; ^9^ Department of Biomedical Engineering Tashkent State Technical University Tashkent Uzbekistan; ^10^ Institute of Food Safety and Environment Monitoring MOE Key Laboratory for Analytical Science of Food Safety and Biology College of Chemistry Fuzhou University Fuzhou China; ^11^ Key Laboratory of Biomedical Imaging Science and System Chinese Academy of Sciences Shenzhen China

**Keywords:** brain tumor, cold atmospheric plasma therapy, radiotherapy

## Abstract

Treating brain tumors requires a nuanced understanding of the brain, a vital and delicate organ. Location, size, tumor type, and surrounding tissue health are crucial in developing treatment plans. This review comprehensively summarizes various treatment options that are available or could be potentially available for brain tumors, including physical therapies (radiotherapy, ablation therapy, photodynamic therapy, tumor‐treating field therapy, and cold atmospheric plasma therapy) and non‐physical therapies (surgical resection, chemotherapy, targeted therapy, and immunotherapy). Mechanisms of action, potential side effects, indications, and latest developments, as well as their limitations, are highlighted. Furthermore, the requirements for personalized, multi‐modal treatment approaches in this rapidly evolving field are discussed, emphasizing the balance between efficacy and patient safety.

## Graphical abstract

The review summarizes various treatment options for brain tumors, focusing on their mechanisms, potential side effects, and recent developments. It emphasizes the need for personalized, multi‐modal treatment approaches in this rapidly evolving field, balancing efficacy and patient safety. The complexity and diversity of brain tumor treatments are highlighted, advocating for ongoing research and technological advancements to improve patient outcomes.

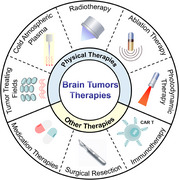

## 1 INTRODUCTION

Brain tumors are abnormal cell growths that occur within or around the brain. In recent years, their frequency has notably increased, particularly in developed countries, making them a prevalent form of malignant tumors.^[^ ^1^ ^]^ These tumors can be either benign (non‐cancerous) or malignant (cancerous), originating either from the brain itself (primary brain tumors) or spreading from other body parts (secondary or metastatic brain tumors). The complexity of brain tumor types, ranging from ependymomas to astrocytomas, reflects the intricate nature of this disease.^[^ ^2^ ^]^ While primary brain tumors may disseminate throughout other regions of the brain or spinal cord, they rarely metastasize to other organs. Unlike primary brain tumors, metastatic brain tumors usually originate in other parts of the body and subsequently spread to the brain, which is more common, with an overall incidence rate of approximately 8.3 per 10,000.^[^ ^3^ ^]^ Metastasis in the brain can occur via hematogenous or lymphatic routes. Any type of cancer can potentially spread to the brain, but lung cancer,^[^ ^4^ ^]^ breast cancer,^[^ ^5^ ^]^ colorectal cancer,^[^ ^6^ ^]^ malignant melanoma,^[^ ^7^ ^]^ and renal cell carcinoma^[^ ^8^ ^]^ are most commonly associated with brain metastases. Brain tumors can cause a range of symptoms, depending on their size, location, and rate of growth.

With the advancement of medical technology, treatment for brain tumors has progressed through various stages. Initially, surgery was the primary method of treating brain tumors. However, due to the complex anatomical location of brain tumors as well as the risks and recurrence rates associated with surgeries, the effectiveness of this approach has been limited. In response, radiation therapy emerged as an effective and non‐invasive alternative in the 1940s, particularly when surgery is unviable or a complete resection of the tumor is not feasible. Though radiotherapy can have side effects like hair loss and skin burn,^[^ ^9^ ^]^ scientific progress from 1930 to 1950 led to developments in radium‐based interstitial irradiation, super‐voltage X‐ray tubes, and electron beam therapy with minimizing side effects,^[^ ^10^ ^]^ making it an important modern treatment option. During the 1960s, chemotherapy began to be utilized in the treatment of brain tumors.^[^ ^11^ ^]^ Compared to surgery and radiotherapy, chemotherapy offers the advantage of killing tumor cells systemically. However, it also comes with a set of side effects, such as nausea, vomiting, and anemia.^[^ ^12^ ^]^ American chemist and chemotherapy pioneer, Farber, successfully synthesized carbonyl compounds that could eliminate tumor cells,^[^ ^13^ ^]^ contributing significantly to the advancement of brain tumor treatment. Over time, a comprehensive treatment model emerged, combining various modalities, including surgery, radiotherapy, chemotherapy, targeted therapy, and immunotherapy. This approach has been proven to improve both cure and survival rates among patients.^[^ ^14^ ^]^ Recent advancements in technology, such as gene sequencing and molecular diagnosis, have paved the way for personalized treatment of brain tumors.^[^ ^15^ ^]^ Precision medicine allows for tailored treatment plans based on individual patient factors such as genotype and molecular characteristics of the tumor, resulting in improved treatment efficacy and safety.

**FIGURE 1:**
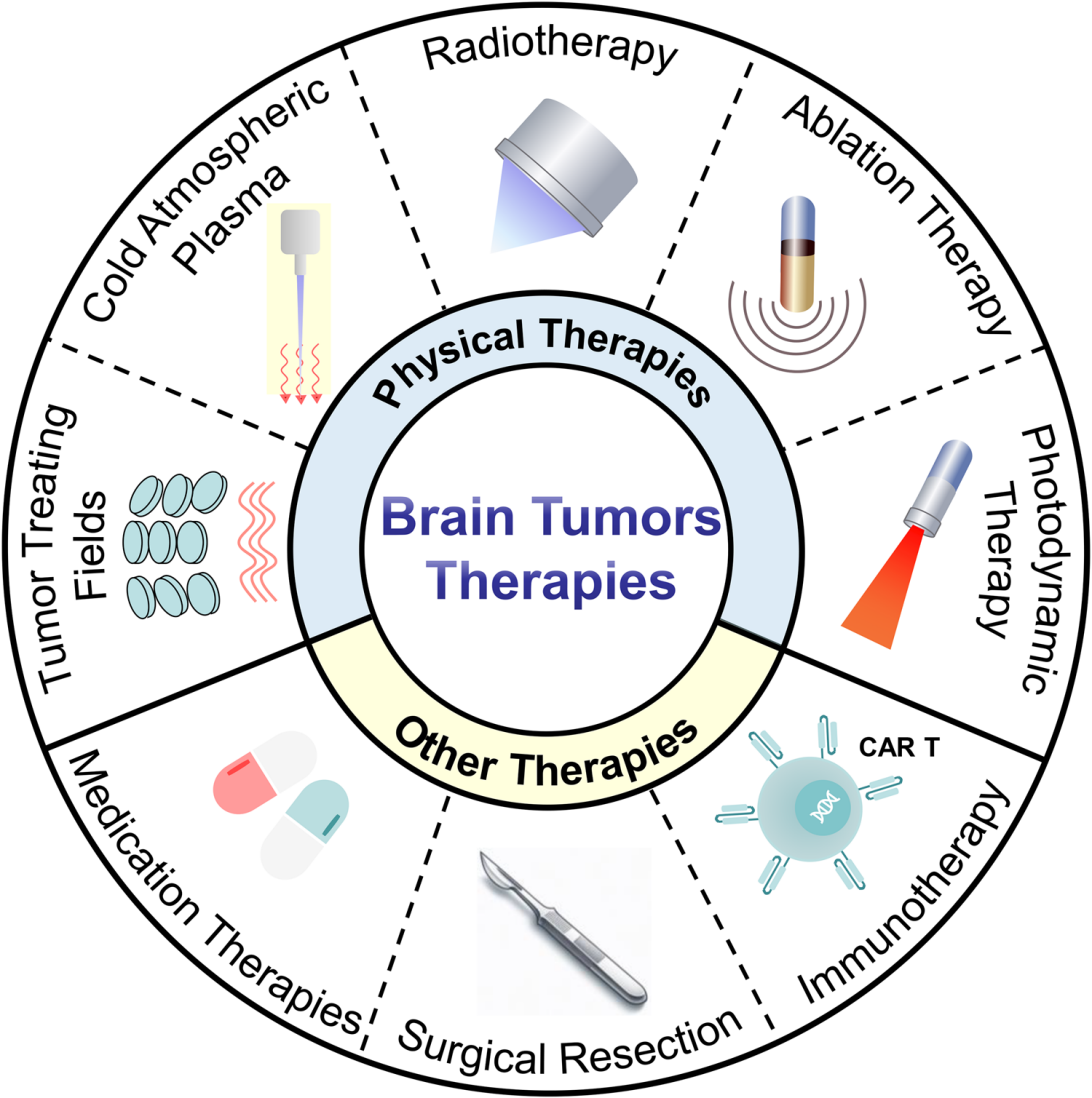
The primary treatment modalities for brain tumors.

Physical therapy, which consists of radiotherapy, ablation therapy, photodynamic therapy (PDT), tumor treating fields (TTFields), and cold atmospheric plasma (CAP), is an essential part of multidisciplinary management for patients with brain tumors, with the primary goal being to minimize brain damage during treatment and improve the patient's quality of life (QOL). Other treatment modalities for brain tumors include surgical resection, chemotherapy, targeted therapy, and immunotherapy (Figure 1). The choice of treatment depends on various factors, including the type and grade of the tumor, its location, and the overall health of the patient. Despite numerous obstacles and challenges, ongoing refinements and advancements have led to an array of improved treatment options, offering greater hope for successful outcomes for patients.

The medical field faces numerous challenges when it comes to treating brain tumors, such as accurately identifying and precisely localizing the tumor within the complex brain structures. This is crucial for determining the most effective treatment approach.^[^ ^16^ ^]^ Additionally, preserving neurological function is of utmost importance during surgical interventions due to the brain's delicate nature. Complete removal of the tumor can be difficult since certain tumors may infiltrate critical brain regions or have irregular shapes, making complete eradication impractical or risky.^[^ ^17^ ^]^ Moreover, managing the side effects of treatment, such as chemotherapy and radiation therapy, is essential to maintain patients' QOL. In addition, the specific nature of brain tissue and the blood‐brain barrier (BBB) makes it more challenging to avoid harm to patients, limiting treatment options.^[^ ^18^ ^]^ However, promising advancements in brain tumor treatment are emerging, such as minimally invasive surgical techniques like laser ablation and stereotactic radiosurgery (SRS), which reduce the risk of damage to healthy tissue. Furthermore, targeted therapies that focus on specific genetic mutations in tumor cells hold the potential for more effective and personalized treatment options with fewer systemic side effects. It is crucial to gain an in‐depth understanding of the underlying mechanisms that contribute to tumor recurrence in order to develop more effective treatment methods while minimizing potential adverse effects. Esmatabadi et al. conducted a comprehensive investigation of potential mechanisms of tumor recurrence and identified three key drivers: cancer stem cells (CSCs), tumor dormancy, and phoenix rising.^[^ ^19^ ^]^ The survival of CSCs is widely recognized as a significant contributor to the resistance of malignant cells to treatment. CSCs have relatively quiescent metabolic activity and are resistant to therapeutic agents through multiple pathways.^[^ ^20^ ^]^ As CSCs are closely linked to tumor initiation, metastasis, and recurrence, targeting them with chemotherapy is a valuable approach for eradicating malignant cells and inhibiting their spread to other parts of the body. Overall, early detection, accurate diagnosis, and a multidisciplinary approach are essential for successful brain tumor treatment. Further research and technological advancements offer hope for improving the outcomes of brain tumor patients in the future.

This review aims to bridge the knowledge gap in understanding the various treatment strategies for brain tumors. It delves into the advancements and challenges faced in treating both primary and metastatic brain tumors, highlighting the nuances between the different types and stages of these tumors. Additionally, we aim to provide a comprehensive overview of the current and emerging treatment modalities, including surgical resection, chemotherapy, targeted therapy, and immunotherapy, and to discuss their effectiveness and limitations. The review also touches upon how cancer types such as lung, breast, colorectal, melanoma, and renal cell carcinoma commonly lead to brain metastases and the implications for treatment strategies.

## 2 RADIOTHERAPY FOR BRAIN TUMORS

**FIGURE 2:**
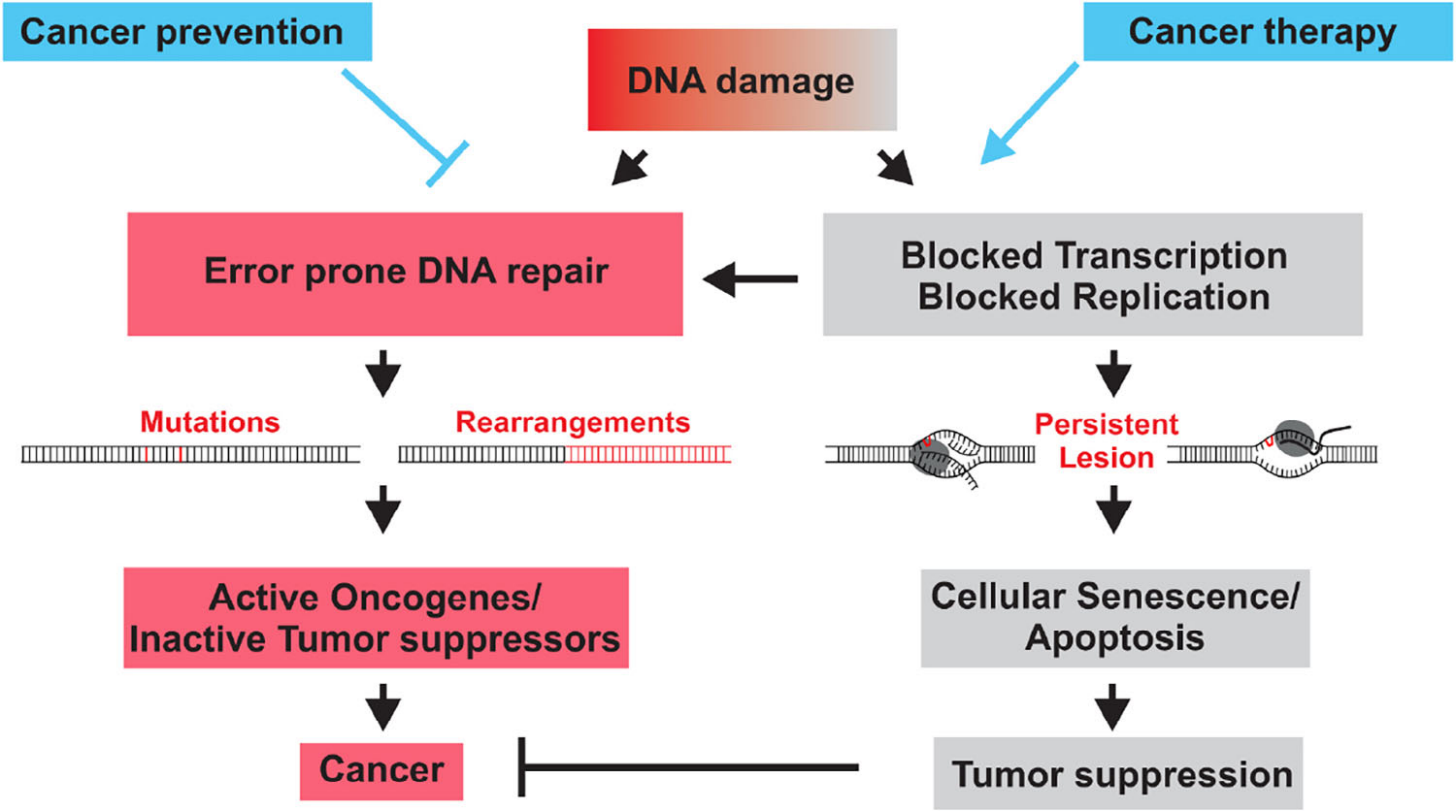
Mechanisms of DNA repair after damage. Reproduced under the terms of the CC‐BY 4.0 license.^[^ ^22^ ^]^ Copyright 2015, Torgovnick and Schumacher.

Radiotherapy is a frequently used treatment for brain tumors that has proven to be effective and non‐invasive, particularly in cases where it is not feasible to conduct surgery or the tumor is inoperable. This therapy can be administrated externally using a linear accelerator or internally by placing radioactive materials directly into the tumor.^[^ ^21^ ^]^ Radiation therapy uses high‐energy X‐rays or other particles to penetrate the skin, skull, and brain tissue. It interacts with the atoms and molecules inside cancer cells, causing DNA damage. When DNA damage persists and disrupts replication or transcription, DNA damage checkpoints are activated, leading to either cell senescence or apoptosis. This leads to the deactivation or removal of impaired cells, consequently impeding or preventing the progression of brain tumors (Figure 2).^[^ ^22^ ^]^ Radiotherapy for brain tumors aims to eradicate cancer cells, reduce the size of the tumor, and alleviate symptoms. It is frequently applied in combination with surgery and chemotherapy.^[^ ^23^ ^]^ As far back as 1954, Chao et al. demonstrated the efficacy of radiation therapy in managing brain metastases.^[^ ^24^ ^]^ The radiation is precisely planned and administered to minimize harm to the healthy brain tissue surrounding the tumor. Although normal brain cells can also be affected by radiation damage, their lower rate of division facilitates better self‐repair as compared to cancer cells. Radiotherapy can lead to side effects such as fatigue, nausea, and long‐term ailments like cognitive dysfunction and radiation‐induced brain damage. The impact on quality of life can vary, with some patients experiencing significant distress due to these side effects. Despite its effectiveness, radiation therapy grapples with the challenge of balancing tumor control against minimizing harm to healthy brain tissue. The risks of radiation necrosis and enduring cognitive impairments are noteworthy, underscoring the necessity for more precise targeting methods and tailored treatment strategies. A study in the “British Journal of Cancer” underscores the significance of comprehending the mechanisms underlying radiation‐induced DNA damage and repair in cancer cells.^[^ ^25^ ^]^ This review highlights the effectiveness of various radiation therapy techniques in treating brain tumors, including whole‐brain radiation therapy, SRS, proton therapy, and brachytherapy. Overall, radiation therapy constitutes an essential treatment modality that impedes cancer cell DNA and halts its proliferation and expansion.

### 2.1 Whole‐brain radiotherapy (WBRT) and stereotactic radiosurgery (SRS)

**FIGURE 3:**
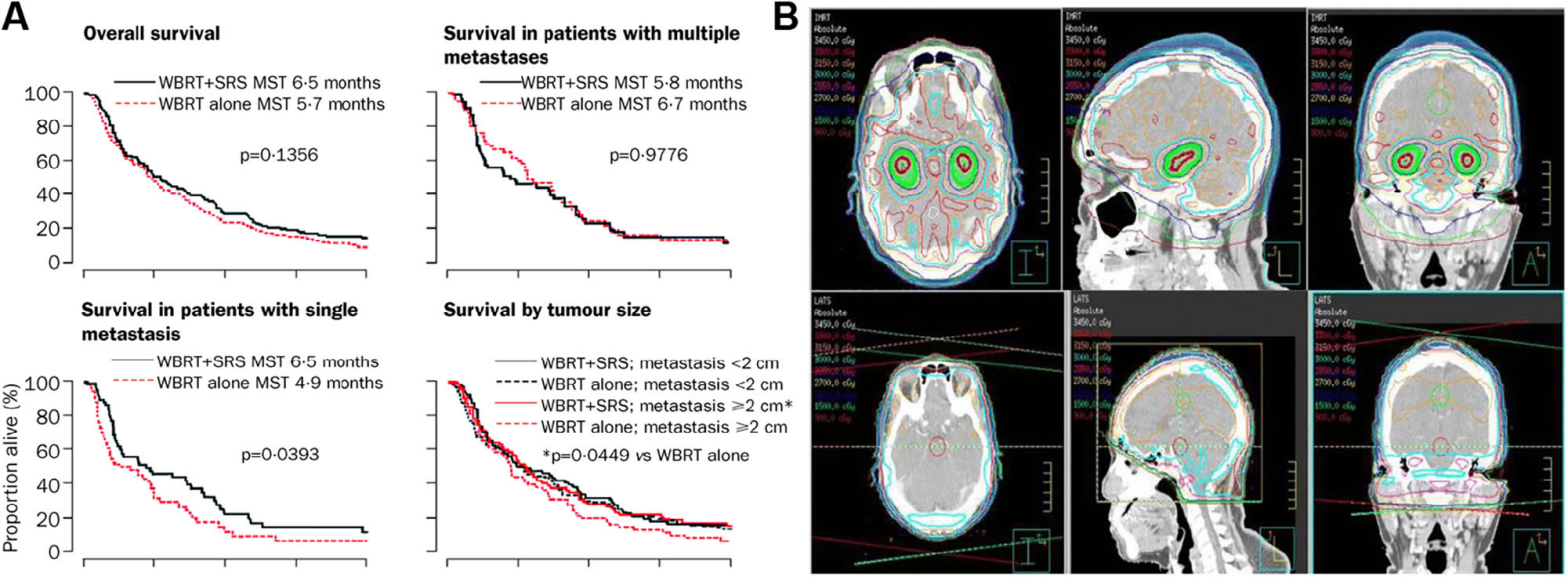
WBRT and SRS for brain tumors. (A) Comparison of treatment outcomes of single WBRT and WBRT + SRS. Reproduced with permission.^[^ ^32^ ^]^ Copyright 2004, Elsevier Ltd. (B) Whole brain radiation therapy that avoids the hippocampus (top three panels, green shaded area) compared with standard whole brain radiation therapy (bottom three panels). Reproduced under the terms of the CC‐BY 4.0 license.^[^ ^34^ ^]^ Copyright 2015, The Authors, published by Springer Nature.

Whole‐brain radiotherapy (WBRT), also referred to as cranial irradiation, is a medical treatment method that entails the use of high‐energy radiation to treat the whole brain. It is typically used to treat several medical conditions, including brain metastases from primary cancers such as lung, breast, or melanoma, as well as certain primary brain tumors.^[^ ^26^ ^]^ The objective of WBRT is to eliminate or manage microscopic cancer cells that may have disseminated throughout the brain, regardless of the absence of apparent tumors. Exposing the whole brain to radiation, it endeavors to decrease the risk of additional tumor growth and avert the formation of fresh lesions.^[^ ^27^ ^]^ During the procedure, the patient reclines in a device that guarantees precise positioning for treatment administration. The radiation is generally administered in daily fractions over several weeks, typically via a linear accelerator or other specialized radiation device. The planning process for treatment involves implementing advanced imaging technologies, such as computed tomography (CT) or magnetic resonance imaging (MRI), to accurately target the brain while reducing radiation exposure to neighboring healthy tissue. The effectiveness of WBRT depends on objective factors, including the size and number of brain metastases or primary tumors, any symptoms experienced by the patient, and their overall condition. To achieve optimal outcomes, WBRT can be combined with other treatment modalities, such as surgery or chemotherapy. Although WBRT maximizes the removal of brain cancer cells, it has become less popular due to concerns about the late toxicity profile caused by WBRT as well as the risk of potential memory loss.^[^ ^28^ ^]^ To overcome these challenges, many researchers have explored ways to enhance WBRT, primarily by combining SRS,^[^ ^29^ ^]^ simultaneous in‐field boost (SIB),^[^ ^30^ ^]^ or chemotherapy.^[^ ^31^ ^]^ Between 1996 and 2001, Andrews and colleagues compared the efficacy of single WBRT with combined WBRT and SRS in treating multiple brain metastases. As shown in Figure 3A, it was found that the combined therapy significantly increased survival rates and reduced tumor size in patients with brain metastases.^[^ ^32^ ^]^ Controlling the dose to preserve the hippocampus and neural stem cells (NSC) can reduce the incidence of WBRT.^[^ ^33^ ^]^ Based on Figure 3B, Dr. Mahadevan used a method known as hippocampal sparing whole‐brain radiation therapy (HS‐WBRT), which entails avoiding the hippocampus during treatment.^[^ ^34^ ^]^ The results indicated that patients who received HS‐WBRT had a lower likelihood of experiencing cognitive decline compared to those who received standard WBRT. Moreover, Dr. Gond claimed that the capacity to spare the hippocampus during WBRT is related to improved memory preservation and QOL.^[^ ^35^ ^]^

**FIGURE 4:**
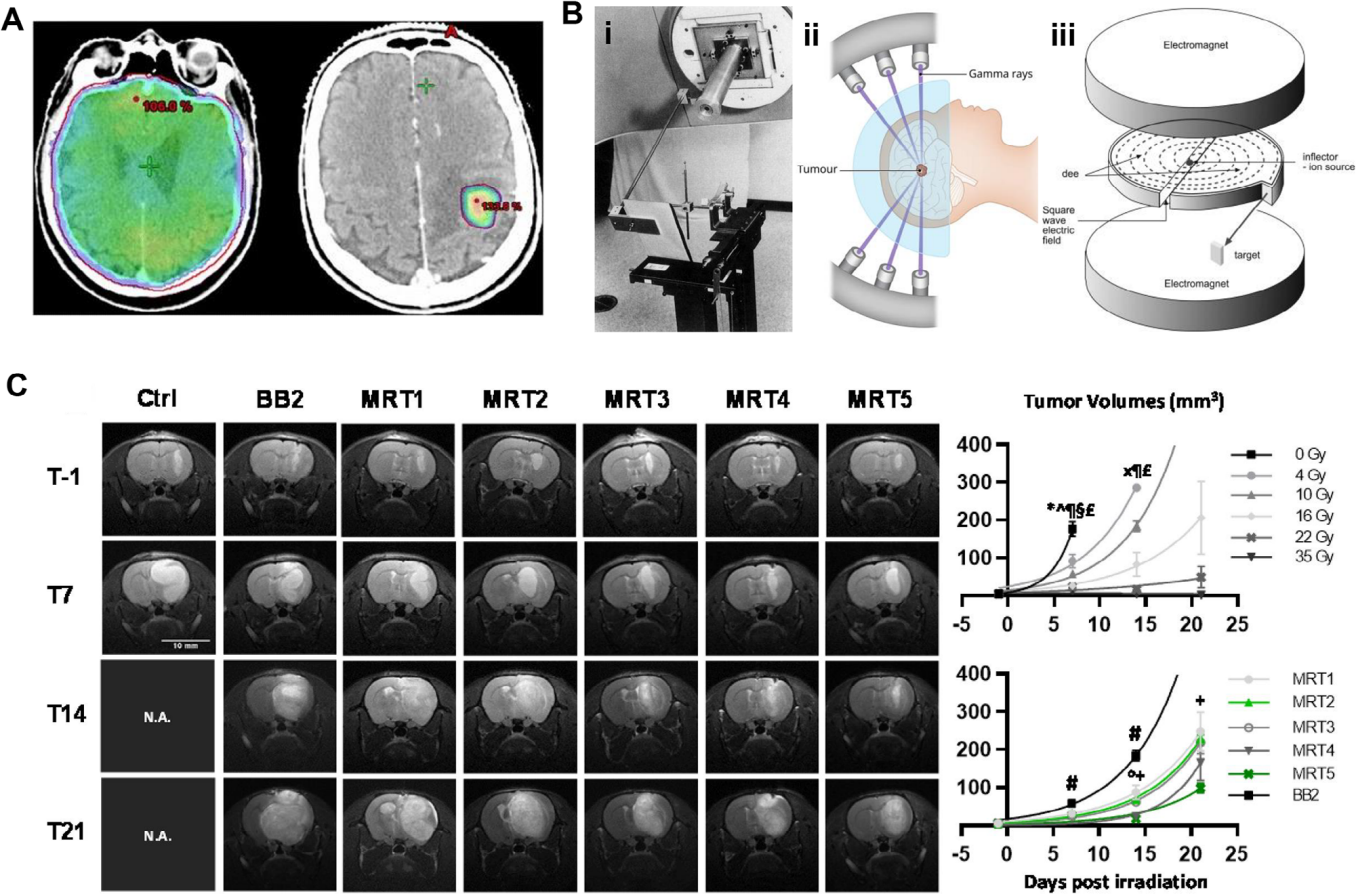
WBRT and SRS for brain tumors. (A) Compares the dose distribution between WBRT and SRS, showing that with WBRT, healthy brain tissue receives a low dose while with SRS, only metastatic tissue receives a high dose. Reproduced under the terms of the CC‐BY 4.0 license.^[^ ^36^ ^]^ Copyright 2015, The Authors, published by Springer Nature. B) Schematic diagram of three different types of radiation delivery systems: LINAC (i). Reproduced with permission.^[^ ^50^ ^]^ Copyright 2002, Elsevier Inc. Gamma knife (ii). Images taken from IHH Healthcare Singapore. Cyclotron (iii). Reproduced with permission.^[^ ^43^ ^]^ Copyright 2006, Elsevier Inc. (C) The influence of five MRT ports on brain tumors was evaluated. The tumor planes were documented on day 1, day 7, day 14, and day 21, and the tumor volumes were compared. Reproduced under the terms of the CC‐BY 4.0 license.^[^ ^47^ ^]^ Copyright 2021, The Authors, published by MDPI.

SRS provides highly precise and targeted radiation using fewer high‐dose treatments than traditional surgery or WBRT, limiting the radiation dose to non‐affected regions of the brain, as illustrated in Figure 4A.^[^ ^36^ ^]^ Furthermore, SRS can achieve a high level of local tumor control with a single treatment, resulting in better QOL for patients.^[^ ^37^ ^]^ Aoyama et al. compared the effectiveness of SRS alone to the WBRT/SRS combination applied to patients with brain metastases after 1 year of treatment. They found that the tumor recurrence rate was significantly lower for patients treated with WBRT and SRS (46.8%) compared to those treated with SRS only (76.4%).^[^ ^38^ ^]^ Moreover, WBRT combined with SRS yielded a higher rate of local control at 12 months (88.7%) compared to SRS alone (72.5%). Given the risk of craniotomy and the serious adverse effects of WBRT, patients prefer SRS. Fractionated stereotactic radiation therapy (FSRT), a refined version of SRS, administering radiation in smaller doses over several treatment sessions, which is advantageous in treating larger tumors or those in critical brain regions.^[^ ^39^ ^]^

SRS utilizes three distinct technologies for delivering radiation: linear accelerators (LINAC), gamma knife, and cyclotron. LINAC employs X‐rays or photons to treat cancerous and non‐cancerous abnormalities. Figure 4B illustrates the precise irradiation of the patient's head using a 6MV LINAC. The G Gamma knife employs either 192 or 201 beams of precisely focused gamma rays on a specific brain region.^[^ ^40^ ^]^ The employment rate of Gamma knife devices is lower compared to that of LINAC devices and is predominantly restricted to small or medium‐sized tumors and intracranial lesions linked to diverse disorders.^[^ ^41^ ^]^ Emmanuel et al., assessed 31 glioblastoma multiforme (GBM) patients who received adjuvant therapy with a gamma knife device for SRS. They determined that individuals who received enhanced GK‐SRS had significantly longer survival times in comparison to those treated with external beam radiation therapy (EBRT) alone.^[^ ^42^ ^]^ Cyclotrons, illustrated in Figure 4B, are used for directing proton beams toward the site of the targeted tumor when treating various cancer types.^[^ ^43^ ^]^ Although cyclotron is expensive and operationally complex, they can effectively protect normal tissue while treating large tumors. Markus et al. reported that patients with early skull‐base tumors treated at the Harvard Cyclotron Laboratory received fractionated 160 MeV proton beam therapy with calculated 5‐ and 10‐year local tumor control rates of 93% and 85%, respectively.^[^ ^44^ ^]^

Compared to the cyclotron, the synchrotron has fewer practical energy limitations, and can produce beams with both high peak intensity and continuous beam emission. The synchrotron accelerator requires an injector, such as a linear accelerator, while the cyclotron generally does not need one. The synchrotron employs varied electric and magnetic fields to accelerate particles to higher energy levels, whereas the cyclotron utilizes a constant magnetic field and frequency electric field.^[^ ^45^ ^]^ Currently, two advanced radiation therapy techniques, synchrotron microbeam radiation therapy (MRT) and synchrotron stereotactic radiation therapy (SSRT), are based on synchrotron accelerator technology. MRT distributes highly parallel X‐ray beams ranging from 50–600 keV into various high‐dose (peak dose) microbeams with regular intervals of low‐dose (valley dose) microbeams. Despite only irradiating ≈12–25% of the area during the beam peak, MRT has proven to be highly effective in treating different tumor types.^[^ ^46^ ^]^ Notably, normal tissue damage is limited during MRT treatment. Dr. Eling and his colleagues utilized MRT to irradiate rats with brain tumors (9LGS) and observed that MRT significantly improves tumor control in all geometric shapes compared to uniform broad‐beam (BB) radiotherapy, as illustrated in Figure 4C, on day 14 and day 21 following infection. Furthermore, the cell proliferation rate decreased after MRT treatment, indicating an extended period of tumor regrowth delay.^[^ ^47^ ^]^ Conversely, SSRT depends on local drug uptake of high‐Z elements in tumors, followed by stereotactic irradiation with 80 keV photons for exclusive dose deposition within the tumor. Dilmian et al. envisioned in vivo dosimetry and static irradiation employing microbeams.^[^ ^48^ ^]^ Although SSRT and MRT differ in principle, their shared characteristics could lead to future combinations. Such merging during clinical trials and the implementation of optimized dosing schedules may unify the synergistic effects of both methods into a novel radiation therapy approach.^[^ ^49^ ^]^ WBRT and SRS are pivotal in managing brain metastases and certain primary brain tumors. While WBRT targets the entire brain to control microscopic cancer spread, SRS provides a precise, focused treatment. Despite their efficacy, concerns about late toxicity and memory loss have led to exploration of methods like hippocampal sparing and combining therapies for better outcomes.

### 2.2 Proton therapy

Compared to X‐ray therapy, proton therapy typically results in less tissue damage and a lower likelihood of secondary tumors.^[^ ^51^ ^]^ This is due to the Bragg peak effect of protons, which means that when a proton beam penetrates tissue, its energy is deposited within a certain depth range and is not released in other areas. While X‐rays continue to pass through and harm normal tissue, protons reach the tumor and stop.^[^ ^52^ ^]^ Proton therapy, a novel form of radiation therapy for treating tumors, is a noninvasive treatment that uses accelerated subatomic particles called protons (positively charged particles) to exterminate cancer cells by inhibiting their division and growth. Proton therapy delivers high‐energy protons from a synchrotron or cyclotron into a patient's tumor through a precisely controlled conformal beam. The energy delivered by protons is adjusted accordingly based on the location, size, and shape of the brain tumor. Because of its precision, proton therapy can effectively target brain cancer with potentially higher doses of radiation and limited damage to surrounding tissue. This approach reduces the risk of new neurological dysfunction, hormone deficiency, or intellectual disability, while maximizing tumor control and minimizing collateral damage and treatment effects.^[^ ^53^ ^]^ As a result, proton therapy delivers less radiation to the non‐targeted brain (defined as the normal brain minus the tumor area) than other radiation treatments. This reduction in dose to normal brain tissue may preserve better overall brain function and reduce the likelihood of future secondary tumors. At the National Accelerator Center (NAC) in the Republic of South Africa, Vernimmen et al. reported the use of protons for stereotaxic treatment of patients with skull base meningiomas, achieving an 89% tumor control rate.^[^ ^52b^ ^]^ Noel et al. performed combined photon and proton radiation therapy on 51 patients with brain tumors at the base of the skull and found a local tumor control rate of 98% and an overall survival rate of 100% at 4 years.^[^ ^54^ ^]^ Wenkel et al. conducted photon and proton beam radiation therapy on 46 patients with partially resected or recurrent brain tumors. The lieutenant followed up for 53 months and found that the 10‐year overall survival rate of the patients was 77%.^[^ ^55^ ^]^ To evaluate the feasibility of proton irradiation, Dennis et al. performed graded proton radiotherapy on 11 patients with low‐grade gliomas (LGG) and found that proton therapy can effectively reduce the surrounding normal tissue in LGG patients’ doses.^[^ ^56^ ^]^ Based on the dosimetric advantages of protons, proton therapy alone or in combination with photons can effectively manage tumors. For patients with large and/or complex‐shaped meningiomas or younger individuals, fractionated proton irradiation may be considered.^[^ ^51^, ^52^ ^]^

**FIGURE 5:**
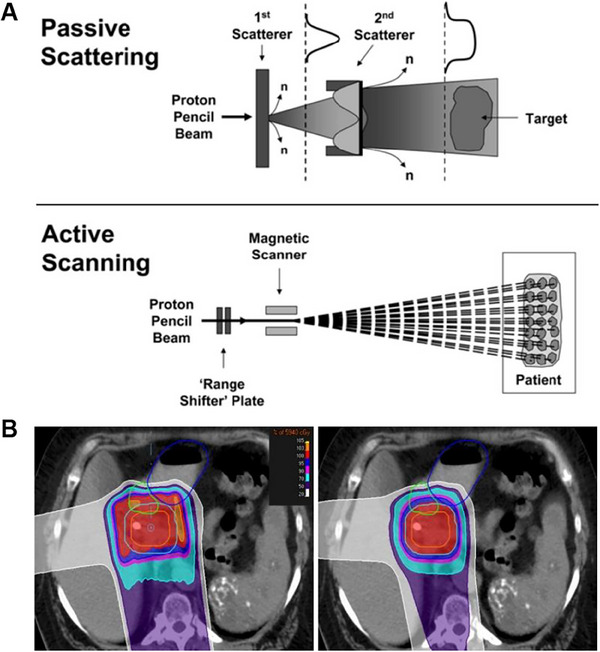
Proton therapy for brain tumors. (A) Two types of proton therapy are used in the treatment of cancer: Active scanning and passive scattering. Reproduced under the terms of the CC BY‐NC‐ND 4.0 license.^[^ ^57^ ^]^ Copyright 2006, The Authors, published by Elsevier. (B) Dose distribution of passive scattering plan (left) and active scanning plan (right). Reproduced under the terms of the CC BY‐NC‐ND 4.0 license.^[^ ^59^ ^]^ Copyright 2018, The Authors, published by AME Publishing Company.

Two types of proton therapy are used in cancer treatment: pencil beam scanning proton therapy (PBSPT) and passive scattering proton therapy (PSPT) (Figure 5A).^[^ ^57^ ^]^ PBSPT is an active scanning technique that delivers a single, narrower beam of protons swept across the tumor magnetically without requiring beam‐shaping equipment. Based on the fact that this technique provides a more precise 3D beam that conforms to the shape and depth of the tumor, PBSPT is often recommended for tumors with complex shapes close to vital organs. PSPT is to diffuse the proton beam into a laterally uniform beam by single or double scattering, which is applied to smaller and larger areas of treatment, with a maximum uniform field diameter of up to 25 cm.^[^ ^58^ ^]^ In a recent study, Chuong evaluated the dosimetric differences of PBSPT in 11 patients with pancreatic cancer who had received PSPT up to 59.4 Gy.^[^ ^59^ ^]^ Compared to passive scattering (PS), the optimized PBSPT showed improved planning target volume coverage and better conformity for treating irregular tumors, as illustrated in Figure 5B. Liao et al. compared PSPT and intensity‐modulated radiotherapy (IMRT) as treatments for non‐small‐cell lung cancer (NSCLC).^[^ ^60^ ^]^ They found that PSPT did not improve lung volume index compared to IMRT, but a positive effect was observed on the heart. No discernible benefit was found regarding radiation pneumonitis (RP) and local failure (LF) after PSPT treatment. Proton therapy, characterized by its precision and reduced collateral damage, represents a significant advancement in brain tumor treatment. Its ability to deliver high doses to tumors while sparing surrounding healthy tissue makes it particularly beneficial for complex cases. Ongoing studies continue to validate its efficacy and explore its potential in combination with other therapies.

### 2.3 Brachytherapy

Brachytherapy, a surgical method, involves placing radioactive implants at tumor removal sites, ideal for non‐metastasized tumors, also known as interstitial brachytherapy. Its localized radiation minimizes adjacent tissue damage. The implants' duration inside the body varies by material; stronger types are removed sooner, while softer ones degrade over time. Table 1 summarizes common implants for different tumors. The use of brachytherapy in treating brain tumors began in the early 20th century. In 1988, Bashir at Massachusetts General Hospital utilized brachytherapy to treat 62 patients who had undergone GBM resection, marking a significant application of this technique in modern medical practice.^[^ ^61^ ^]^ In addition, they evaluated patterns of tumor regeneration in both the adjacent and non‐adjacent areas of the resection site. Results indicated a 51.6% and 43.5% rate of tumor regeneration in the resection site and adjacent areas, respectively. Consequently, the radiation fields for interstitial brachytherapy must encompass the resection site entirely to effectively treat glioblastoma.

**TABLE 1:** Representative radioactive implants.

Name	Type of the cancer	Describe	Examples
Iodine‐125 (I‐125)	Prostate cancer, brain tumors, and eye tumors	Its half‐life is 60 days, and the gamma rays it emits can penetrate tissue but not skin.	[64]
Palladium‐103 (Pd‐103)	Prostate cancer	Similar to I‐125, because of its mobility, its radioactive effect is more limited to surrounding tissue.	[65]
Vanadium‐48 (V‐48)	Deep‐seated tumors and head and neck tumors	Its half‐life is 16.6 days, providing faster radiation therapy effects than I‐125	[66]
Silver‐107 (Ag‐107)	Lung cancer, pancreatic cancer, liver cancer, and prostate cancer	Its half‐life is 7 hours, and the gamma rays it emits can effectively kill cancer cells	[67]
Rhodium‐106 (Rh‐106)	Eye tumor and oral cancer	Its half‐life is 30.2 days, and its radiation intensity is moderate, effectively killing cancer cells during local treatment	[68]
Yttrium‐90 (Y‐90)	Liver cancer and lymphoma	Its half‐life is 64 hours, and it can effectively kill tumor cells, but because of its high radioactivity, its use must be handled with caution	[69]
Boron‐10 (B‐10)	Brain tumors, head and neck tumors, and skin cancer	B‐10 can promote neutron capture and release high‐energy particles to kill cancer cells	[70]

**FIGURE 6:**
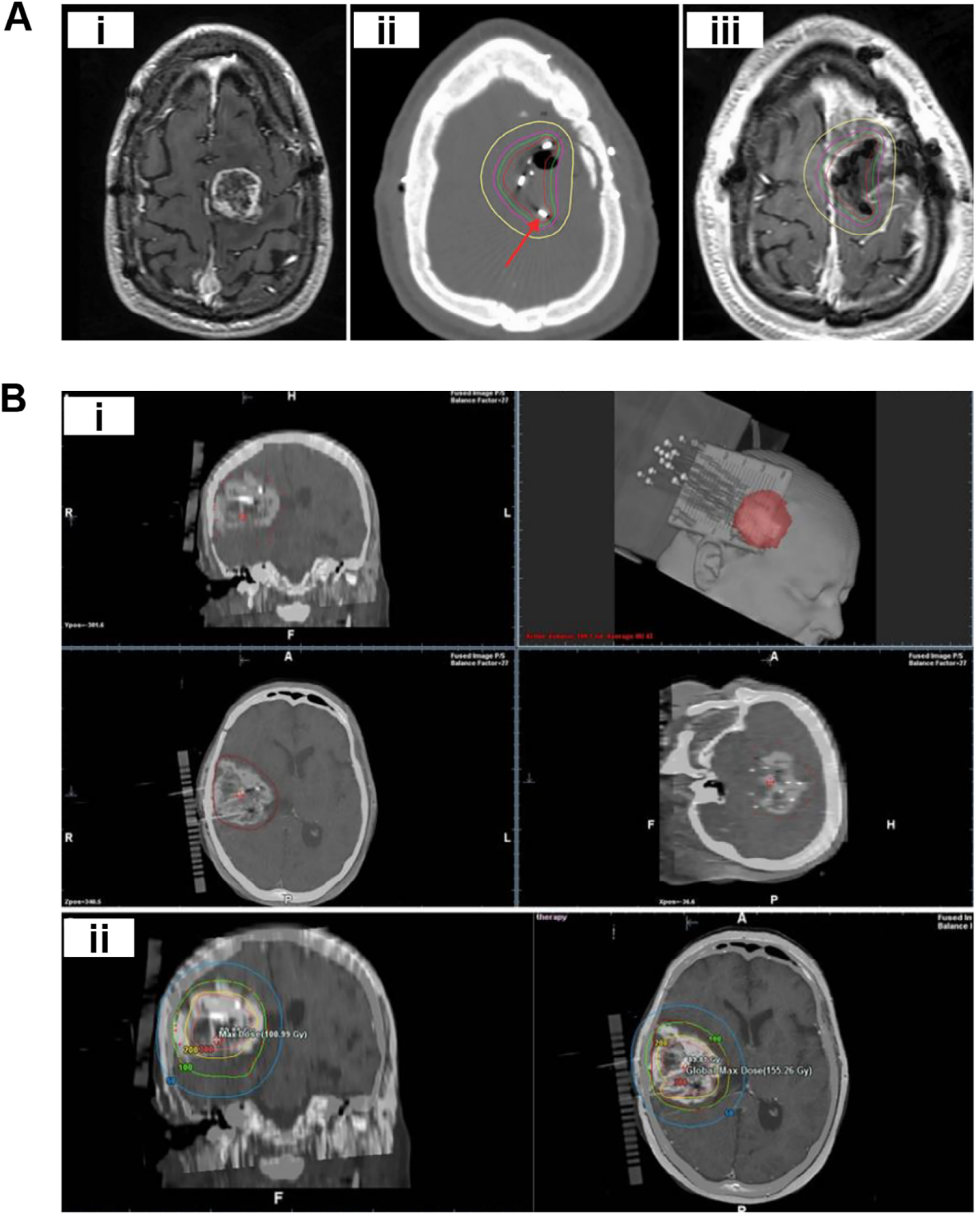
Brachytherapy for brain tumors. (A) Preoperative axial postcontrast T1‐weighted MR image (i). Postoperative axial CT image showing dosimetry with 30‐ (yellow), 60‐ (magenta), 80‐ (green), and 120‐Gy (red) isodose lines, and Cs‐131 seeds (arrow) (ii). Postoperative axial postcontrast T1‐weighted MR image with isodose lines as described in (B) and seeds appearing as small areas of signal void (iii). Reproduced under the terms of the CC‐BY 4.0 license.^[^ ^63^ ^]^ Copyright 2022, The Authors, published by Springer. (B) Interstitial HDR brachytherapy for the treatment of glioblastoma: Multi‐plane three‐dimensional view of 13 in situ close‐range treatment catheters (i). CT‐guided image of the overlapping dose distribution of the uniform implants (ii). Reproduced with permission.^[^ ^21^ ^]^ Copyright 2018, Springer Nature.

Brachytherapy can be classified into three categories based on the dosage rate, that is, low dose rate (LDR, 0.4 to 2 Gy h^−1^), medium dose rate (MDR, 2 to 12 Gy h^−1^) high dose rate (HDR, more than 12 Gy h^−1^).^[^ ^62^ ^]^ A permanent or temporary radiation source is surgically placed in a catheter inside the tumor in case of LDR. David recently performed permanent intracranial brachytherapy (R + BT) with a median radiation dosage of 63 Gy in patients with recurrent and previously irradiated meningiomas using a combination of maximum safe resection and adjuvant radiation.^[^ ^63^ ^]^ Figure 6A displays preoperative and postoperative axially enhanced T1‐weighted MR images. The isodose line revealed a small signal cavity. The utilization of Cs‐131 sources for R + BT resulted in a median overall survival period of 26 months, demonstrating favorable treatment tolerance. For HDR brachytherapy, the GliaSite device (IsoRay Corp., Richland, WA) can be used to insert a device with a highly active radioactive source inside a temporary uniform catheter within the tumor. Furthermore, the double‐walled balloon section of the GliaSite catheter is positioned in the excision cavity. HDR brachytherapy is frequently combined with external beam radiotherapy (EBRT) to treat intermediate‐ and high‐risk diseases, with a disease control rate exceeding 90%. As illustrated in Figure 6B, Chatzikonstantinou et al. reported the results of their study on the efficacy of CT‐guided interstitial HDR brachytherapy for the treatment of recurrent GBM.^[^ ^21^ ^]^ Over a median follow‐up of 9 months, the study demonstrated a median overall survival of 9.2 months after the brachytherapy treatment. The results suggest that HDR brachytherapy is a highly effective treatment option with minimal risk of excessive toxicity. Intracavitary brachytherapy effectively controlled the morbidity, making it a reasonable alternative to craniotomy. Brachytherapy, involving the placement of radioactive implants at tumor sites, offers targeted radiation treatment, especially beneficial for localized tumors. Its minimal damage to adjacent tissues and historical evolution reflects its significance in the treatment spectrum, although its application must be carefully considered based on tumor characteristics. Radiotherapy plays a crucial role in the management of brain tumors, whether as a primary or adjuvant therapy after surgery or chemotherapy. It is a valuable tool in combating brain tumors, providing optimism for improved outcomes and prolonged survival for patients. Advancements in radiotherapy techniques, such as MRT, SRS, and proton therapy, have enhanced treatment accuracy and efficacy while minimizing side effects. These technologies enable more precise delivery of radiation, preserve healthy tissue, and reduce collateral damage.

## 3 ABLATION FOR BRAIN TUMORS

Tumor ablation is a non‐invasive or minimally invasive method for treating tumors that destroys cancer cells using either heat or cold. This technique includes common methods such as microwave, laser interstitial thermal therapy, and cryoablation, which are generally well‐tolerated. Side effects are usually localized and may include pain or discomfort at the treatment site. These therapies typically have a lesser impact on overall quality of life compared to more invasive treatments. Ablation is ideal for certain types of localized tumors in the liver, lung, brain, kidney, bone, and other sites. It can effectively control the growth and spread of tumors and has good efficacy for patients who are not suitable for surgery.

**FIGURE 7:**
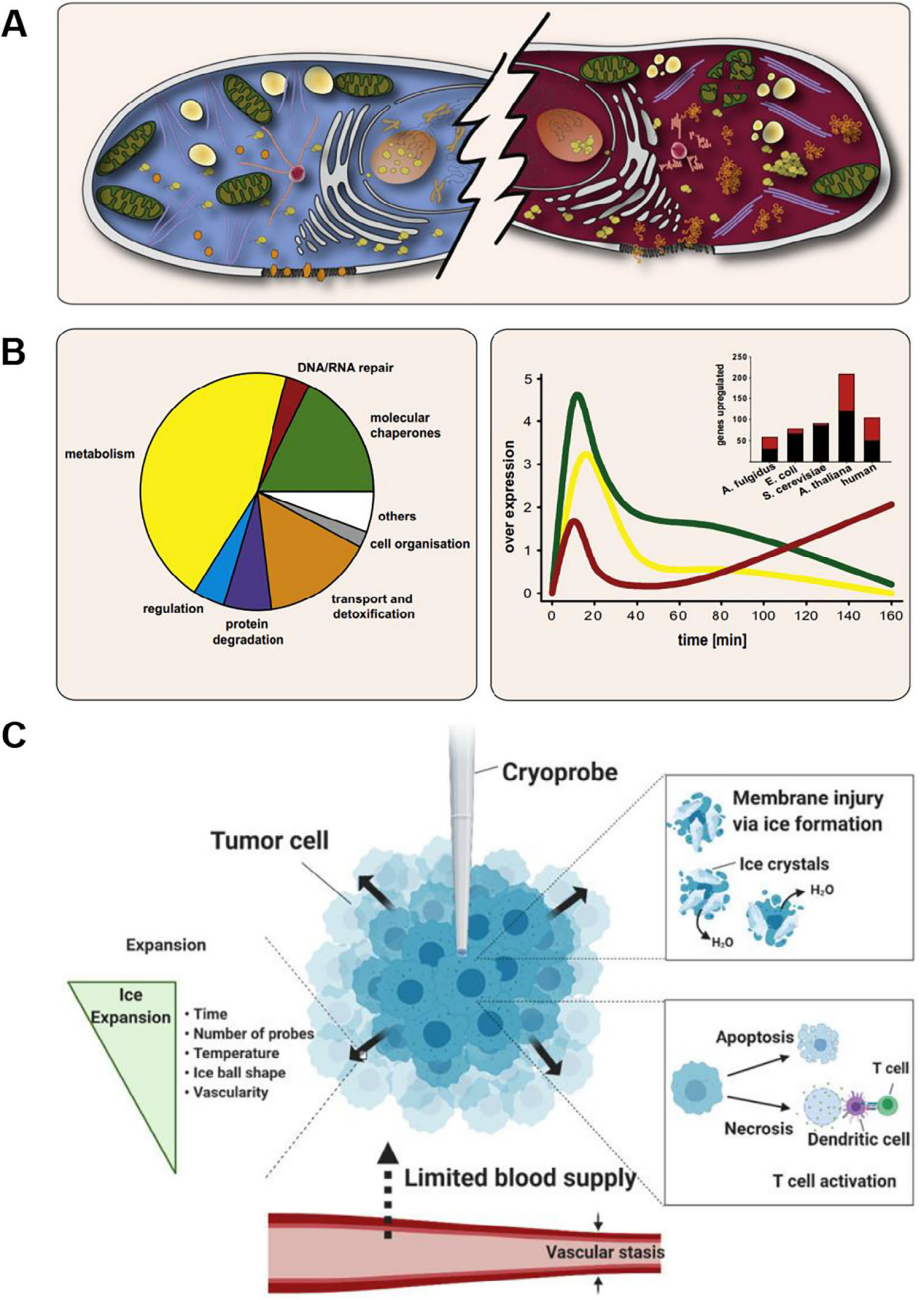
Ablation therapy for brain tumors. (A) Effects of heat shock on the organization of the eukaryotic cell. B) The heat shock proteins. Reproduced under the terms of the CC‐BY 4.0 license.^[^ ^71^ ^]^ Copyright 2010, The authors, published by Elsevier Inc. C) Mechanisms of cryoablation. Reproduced under the terms of the CC‐BY 4.0 license.^[^ ^72^ ^]^ Copyright 2022, The Authors, published by Ivyspring International Publisher.

Hyperthermia achieves acute coagulative necrosis through the generation of high temperatures in the tissue (Figure 7A,B).^[^ ^71^ ^]^ At 41°C, blood vessels dilate and blood flow increases, initiating the heat shock response. This response is a swift gene expression procedure that aims to counteract heat‐induced damage. Heat shock proteins are produced during this response, which can boost the heat resistance of tissues that have survived the initial heat injury. Irreversible damage occurs when the temperature rises between 42 and 46°C, resulting in significant necrosis after approximately 10 min. Within this temperature range, cells and tissues sustain severe thermal injuries, causing irreversible structural and functional changes. As the temperature increases between 46 and 52°C, the combined effects of microvascular thrombosis, ischemia, and hypoxia accelerate cell death. Cells endure more severe damage within a specific temperature range, hastening the process of cell death. When the temperature exceeds 60°C, proteins undergo denaturation, and the cell membrane dissolves, leading to nearly immediate cell death. Hyperthermia achieves cell and tissue necrosis by heating the target tissue within a certain temperature range, triggering the heat shock response, permanent changes in cell structure and function, thrombosis, and hypoxia. This treatment method effectively eradicates tumor cells, thereby diminishing or eradicating tumors.

Cryoablation achieves cell death through various mechanisms (Figure 7C).^[^ ^72^ ^]^ Temperatures below −20°C induce the formation of ice crystals within cells during the cryoablation process, leading to physical harm to the cell membrane. This membrane disruption causes the release of cell contents, including damage‐associated molecular patterns and tumor‐specific antigens, potentially triggering an immune response. Additionally, cryoablation activates cellular stress responses and the intracellular cell death pathway, which is a protective mechanism regulating cell survival or death decisions. The procedure also disrupts mitochondrial function, potentially leading to cell apoptosis. After thawing, apoptotic pathways are further activated, promoting cell death. Moreover, cryoablation causes stagnant blood flow within the frozen tissue, depriving the tumor of its blood supply and exacerbating cell damage. Cryoablation causes cell death through the formation of ice crystals, resulting in membrane damage, the initiation of cellular stress responses, necrosis, and apoptosis cascades, and increased cellular injury. It may also stimulate the immune system to enhance immune responses against tumors.

### 3.1 Microwave

Microwave is a type of electromagnetic radiation with a frequency typically ranging from 1 to 300 GHz. They possess a relatively short wavelength (between 1 mm and 1 m, which is 1000 times that of visible light), hence the name “microwave.” Microwaves have several applications, such as communication, radar, wireless transmission, and cooking, which is one of the most commonly known applications. Microwave technology has been proven to be an effective tool for treating tumors. The application of microwave energy has two main methods: microwave ablation (MWA) and microwave hyperthermia (MWH). MWA involves delivering microwave energy directly to the tumor area, which heats and eventually destroys the tumor cells. MWH, also known as microwave radiofrequency therapy, utilizes the heat generated by microwave energy to treat tumors without the need for invasive procedures. Both MWA and MWH exploit the thermal effects of microwave energy to effectively eradicate tumor cells. They have shown potential for treating tumors, but their specific selection and application depend on the patient's condition and the doctor's advice.

#### 3.1.1 Microwave ablation (MWA)

**FIGURE 8:**
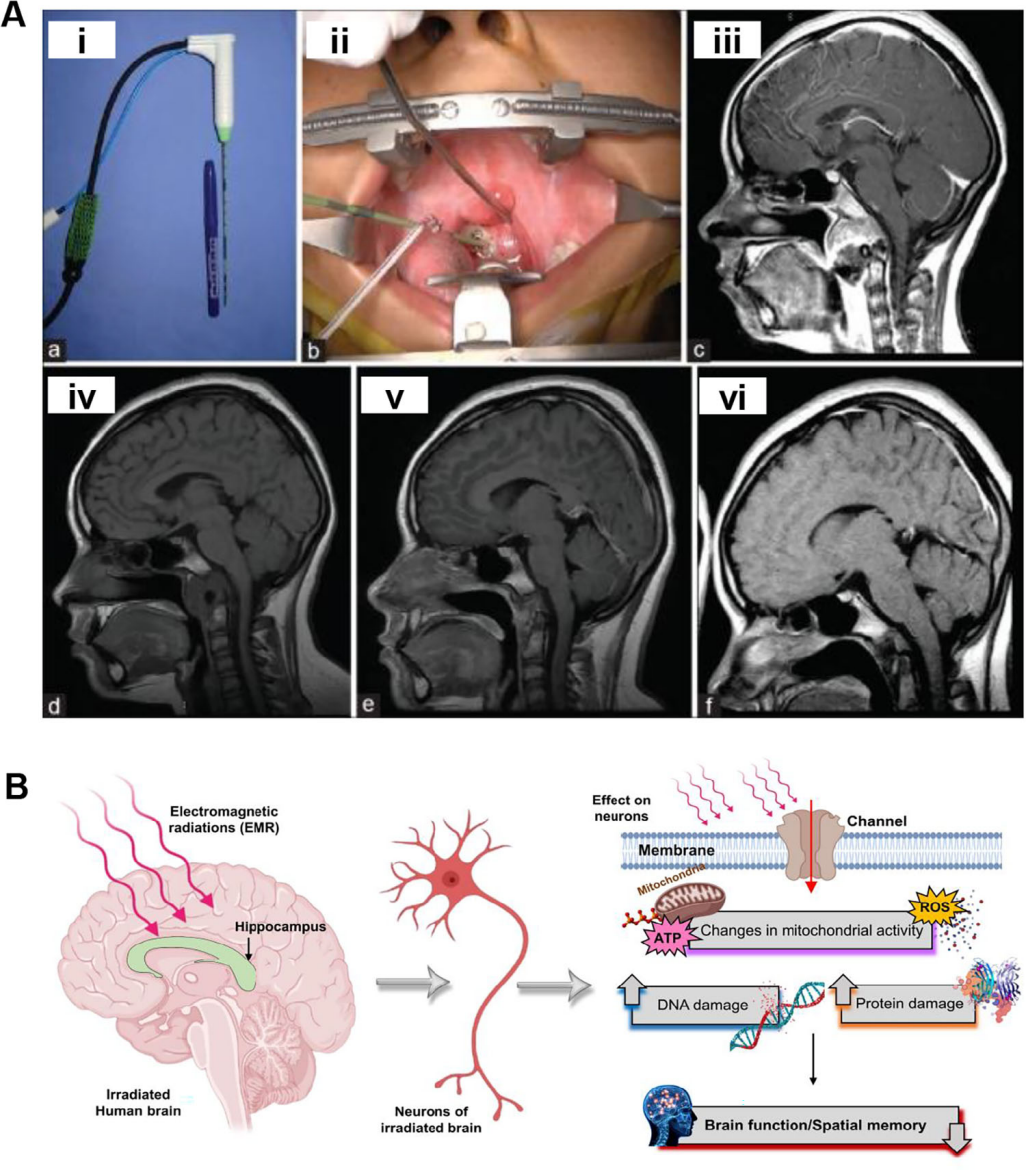
Microwave ablation for brain tumors. (A) MWA was utilized directly on the tumor via a transoral route under general anesthesia, with fluoroscopy guiding the ablation probe precisely to the targeted spot. A thin microwave antenna (i). The ablation antenna was inserted transorally targeting the lesion (ii). Preablation sagittal T1‐enhanced MRI depicted a clivus lesion (iii). An MRI eight months post‐ablation showed enhanced sagittal T1 imaging (iv). A further sagittal T1‐enhanced image was taken 14 months after the treatment (v). A sagittal MRI enhanced in T1, 30 months post‐ablation, illustrated continued lesion regression (vi). Reproduced under the terms of the CC BY‐NC‐SA 4.0 license.^[^ ^77^ ^]^ Copyright 2019, The Authors, published by Scientific Scholar LLC. B) Effects of microwave radiation on the brain. Reproduced under the terms of the CC‐BY 4.0 license.^[^ ^78^ ^]^ Copyright 2022, The Authors, published by MDPI.

MWA is a minimally invasive technique employed to selectively eradicate tumors by inducing high temperatures within the tumor tissue. The procedure commences with precise targeting of the tumor or affected tissue, using sophisticated imaging modalities such as CT scans or ultrasound. This allows for the accurate placement of a specialized microwave antenna, also known as a probe or an applicator, directly into the tumor or adjacent to it. Once the antenna is positioned, it delivers microwave energy at specific frequencies that effectively penetrate and interact with the water molecules present in the tumor tissue. This interaction quickly heats and agitates the water molecules, ultimately generating high temperatures within the tumor.^[^ ^73^ ^]^ Microwave energy is carefully controlled and delivered precisely to ensure effective tumor ablation while minimizing damage to adjacent healthy tissues. Temperature sensors or probes are used during the procedure to monitor the temperature both within the tumor and the surrounding tissues. This monitoring enables the medical team to adjust the energy delivery as necessary for achieving and sustaining the desired temperature range, typically between 50 and 60°C, ensuring efficient elimination of the tumor cells. The high temperatures generated by the microwave energy stimulate coagulative necrosis, a process that irreversibly damages and destroys the tumor cells. Eventually, the body's natural healing processes remove the necrotic tissue, leading to the gradual elimination of the tumor. The potential benefits of microwave technology include faster ablation times, broader ablation regions, and improved performance in highly conductive tissues such as the liver.^[^ ^74^ ^]^ According to the literature, the overall survival of patients with hepatocellular carcinoma (HCC) who underwent MWA therapy was reported to be 22 months for focal lesions larger than 3 cm and 50 months for focal lesions smaller than 3 cm, which is more effective compared to other ablative treatment modalities.^[^ ^75^ ^]^ MWA has demonstrated efficacy as a stand‐alone treatment option for small, localized tumors or as adjunctive therapy in combination with other treatment modalities, such as surgery or chemotherapy.^[^ ^76^ ^]^ Figure 8A illustrates the use of MWA on a tumor via a transoral route under general anesthesia, with fluoroscopy aiding in positioning the ablation probe.^[^ ^77^ ^]^ Following treatment, symptoms improved immediately, and magnetic resonance imaging showed a significant reduction in tumor size. A second session after 8 months led to the tumor's complete elimination by the one‐year mark. In general, MWA is a precise procedure that harnesses microwave energy to generate high temperatures within tumors. This technique offers effective tumor destruction while minimizing damage to healthy tissues through careful targeting and heat delivery, making it a valuable option for treating solid tumors in different organs.

MWA is an interventional treatment method that delivers microwave energy directly to the tumor area, heating and eventually destroying the tumor cells. While effective, MWA requires careful consideration of safety, particularly when used in the brain, where the brain is highly sensitive to electromagnetic radiation (EMR) exposure. As shown in Figure 8B, microwave therapy may induce the induction of electrical currents in brain tissue, disrupt the BBB, and activate proteins and enzymes, which can lead to changes in brain function and behavior.^[^ ^78^ ^]^ Moreover, microwave therapy may result in pain, burns, and skin reactions, as well as neurological symptoms like dizziness, headache, and impaired vision. However, with appropriate treatment settings, the risk of these side effects is minimal. Further research is necessary to determine both the safety and effectiveness of microwave therapy. In most microwave therapies, the microwave heating time or power decreases, possibly due to thermal damage to the tumor vascular system caused by microwaves. To mitigate these risks, advanced imaging techniques are used for precise targeting and temperature monitoring during the procedure. Microwave therapy, whether used alone or in conjunction with radiotherapy and chemotherapy, shows great promise as a treatment option for malignant tumors.

#### 3.1.2 Microwave hyperthermia (MWH)

**FIGURE 9:**
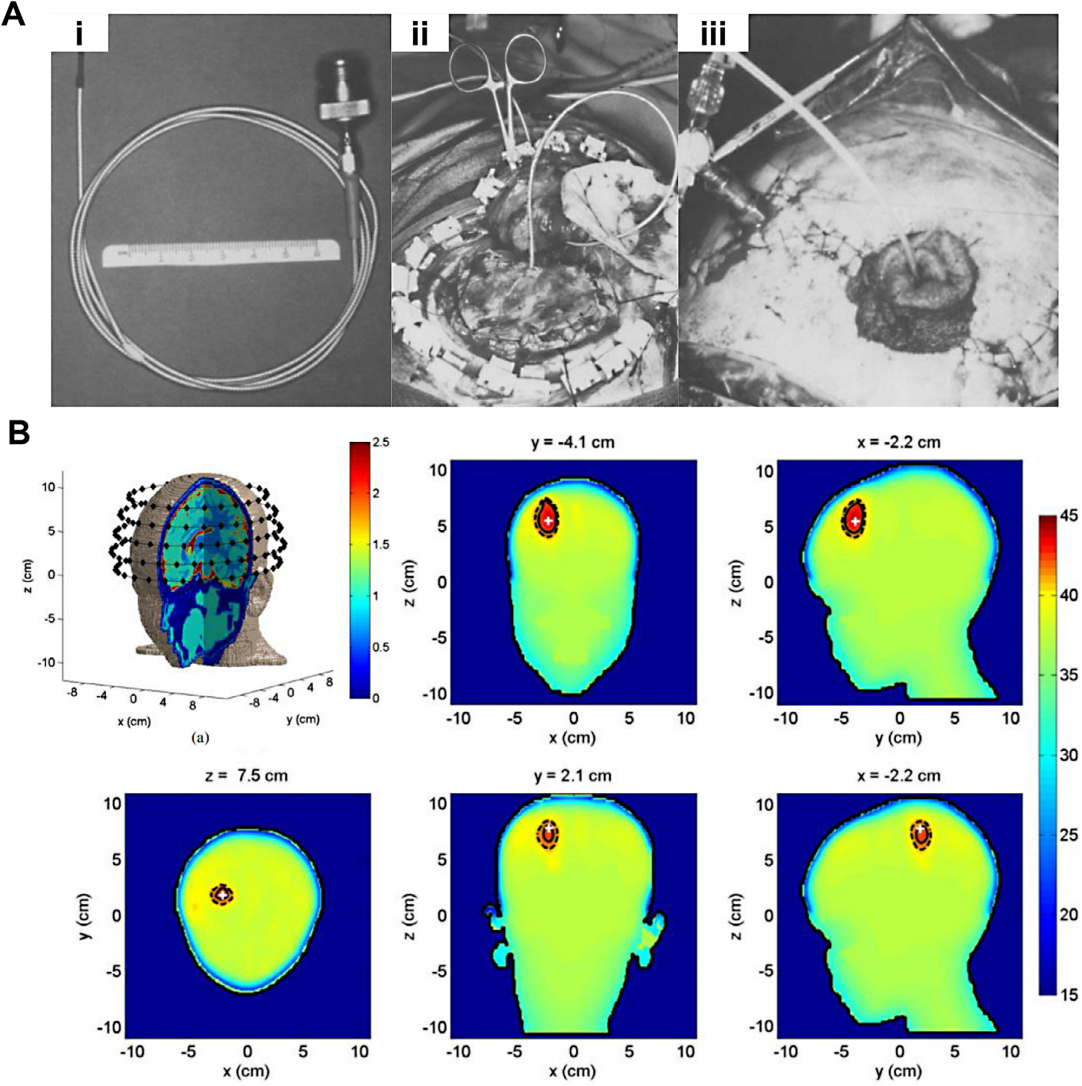
Microwave hyperthermia for brain tumors. (A) Schematic diagram of a microwave applicator for brain tumor treatment: Microwave applicator (i). The applicator and central thermosensitive resistance wire pass through the skull and enter the lesion location (ii). Schematic diagram showing the successful implantation of the applicator (iii). Reproduced with permission.^[^ ^79^ ^]^ Copyright 1983, Springer Nature. (B) Schematic diagram of the array‐based transmit beamformer used for non‐invasive hyperthermia treatment of pediatric brain tumors and the steady‐state temperature distribution of the patient's brain with the highest temperature location. White crosses indicate projections on each orthogonal section. Solid lines represent the heating zone (*T* > 42°C), and dashed lines represent the 41°C contours. Reproduced with permission.^[^ ^85^ ^]^ Copyright 2011, IOP Publishing Ltd.

MWH is a treatment modality that uses high‐frequency electromagnetic waves to thermally stimulate the tissues. It is commonly utilized as an adjunctive therapy along with other treatments, such as radiation therapy or chemotherapy. In conventional MWH, a specialized microwave applicator device is used to generate and deliver electromagnetic waves to the targeted area, as shown in Figure 9A.^[^ ^79^ ^]^ These waves penetrate the tissues and interact with the water molecules present in the cells. As the waves interact with the water molecules, they cause rapid molecular movement and generate heat within the tissues. The aim is to raise the temperature of the targeted area to a therapeutic range, typically between 40 and 45°C. This controlled heating can enhance the effectiveness of other treatments, such as radiation therapy, by sensitizing tumor cells to radiation or enhancing drug delivery in the case of chemotherapy. Heat has several beneficial effects on the body. It can directly damage or kill cancer cells through protein denaturation and disruption of cellular structures. Heat can also improve the effectiveness of radiation therapy by increasing blood flow and oxygenation to the treated area, as oxygen is required for the generation of free radicals that damage DNA in cancer cells. Moreover, heat enhances the immune response, making it more effective in targeting and eliminating cancer cells.^[^ ^80^ ^]^

The safety of MWH in brain tumor treatment involves ensuring the heat is confined to the tumor area and avoiding excessive temperatures that could harm healthy brain tissue. Therefore, the MWH procedure necessitates meticulous planning and monitoring to achieve the desired level of heating in the targeted region while minimizing negative effects on surrounding healthy tissue. Temperature sensors are used to monitor the temperature level during the treatment, ensuring that the therapeutic temperature range is reached and maintained throughout the procedure. In the 1990s, Gottlieb used microscopy to fabricate interstitial hyperthermia applicators with submillimeter diameters that are sturdy and sufficiently potent for employment in patients, rendering them appropriate for the percutaneous treatment of deep‐seated tumors, as well as the intraoperative approach.^[^ ^81^ ^]^ Advancements in ablation technology have led to the development of internally cooled antennas, significantly enhancing the efficiency of the procedure. Kuang et al. developed new internally cooled antennas for ablation using a 14‐gauge shaft with 4°C saline cooling. This innovation maintained lower shaft temperatures during ablation and resulted in more spherical ablation zones compared to uncooled antennas, which caused heat to extend up the shaft, altering the ablation shape. The cooling technique also enabled larger ablation areas and prevented skin burns, enhancing the safety and effectiveness of percutaneous ablation treatments.^[^ ^82^ ^]^

Microwave technology is being investigated as a potential non‐invasive treatment option for solid tumors, such as brain cancer. In a study by Winter et al., twelve patients with advanced‐stage malignant brain tumors, unresponsive to conventional therapies, underwent microwave thermotherapy.^[^ ^83^ ^]^ The experimental outcomes indicated that 75% of the patients' tumors were controlled and exhibited a good clinical response. It was also demonstrated that repeated microwave treatments do not lead to toxicity. Rodrigues and his team introduced a new type of annular phased array applicator with 72 antennas, capable of generating 915 MHz, aimed at targeted heating of brain tumors.^[^ ^84^ ^]^ The results of the study proved the feasibility of using a microwave applicator. The provision of a dedicated, noninvasive brain antenna could considerably enhance the clinical outcome of radiotherapy treatment. Moreover, Burfeindt performed finite‐difference time‐domain simulations on an MRI‐derived child head model to evaluate the effectiveness of array‐based transmission beamforming for non‐invasive thermal therapy of pediatric brain tumors (Figure 9B).^[^ ^85^ ^]^ The results demonstrated that the beamforming created a focused heating zone in the head model at the focal point, avoiding heavy encasement by cerebrospinal fluid. In a study of six patients with recurrent GBM, intracavitary hyperthermia therapy (60 min each) following tumor resection showed a median progression‐free survival of 6.25 months and an overall survival of 8.15 months. The treatment induced a strong inflammatory and antitumoral immune response, suggesting potential benefits for recurrent GBM patients. These promising findings suggest that further research and development are warranted for this technology. MWH has shown promise in the treatment of various types of tumors using minimally invasive approaches, which enable targeted treatment with fewer side effects and shorter recovery times compared to traditional surgery. While MWA and MWH show potential in treating brain tumors, their safety, particularly regarding thermal effects on sensitive brain structures, is paramount. Overall, MWH is a technique that harnesses high‐frequency electromagnetic waves to selectively heat tissues, offering potential benefits for cancer treatment when used alongside other modalities like radiation therapy or chemotherapy.

### 3.2 Laser‐induced thermal therapy (LITT)

**FIGURE 10:**
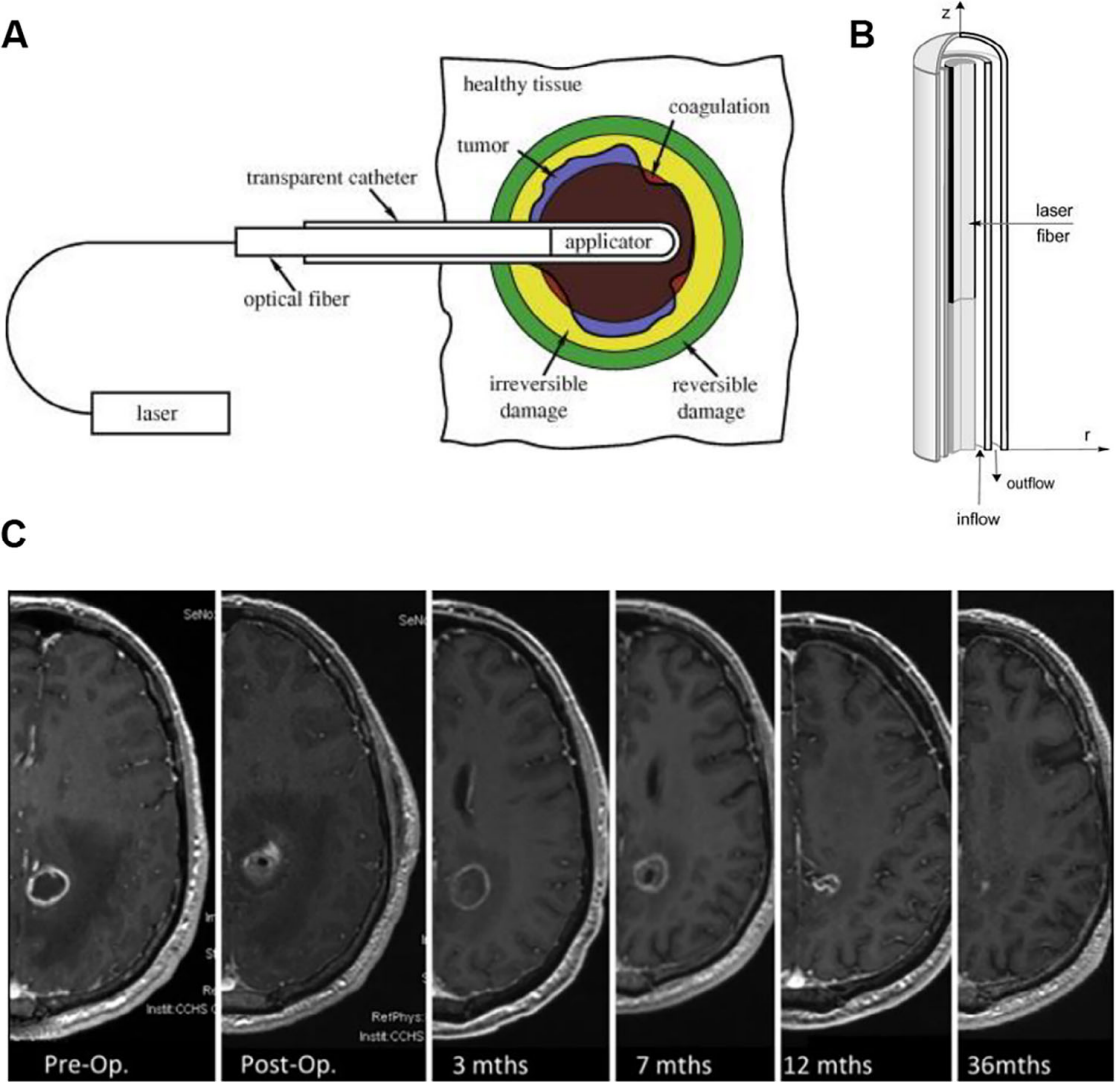
LITT for brain tumors. (A) Schematic diagram of LITT. (B) Schematic of the applicator device. Reproduced with permission.^[^ ^86^ ^]^ Copyright 2010, Elsevier Inc. (C) The T1‐weighted MRI image of a typical case of GBM located in the left parietal lobe, treated with LITT, revealed a significant reduction in the tumor mass. Reproduced under the terms of the CC BY‐NC‐ND 4.0 license.^[^ ^89^ ^]^ Copyright 2015, The Authors, published by the Journal of Neurosurgery Publishing Group.

Laser‐induced thermal therapy (LITT) is a tumor treatment method that uses a laser beam to directly irradiate tumor tissue. The laser energy heats the tumor tissue, ultimately leading to destruction and inactivation. LITT includes a laser light source, a fiber optic guide, and a probe, as illustrated in Figure 10A,B.^[^ ^86^ ^]^ The laser produces a high‐energy laser beam, which is guided by a fiber optic guide into a probe placed on tumor tissue in the patient's body. The laser's optical energy is converted into thermal energy, causing the tumor tissue to heat up when irradiated by the beam and producing a thermal effect. When tissue reaches a certain temperature range, it triggers a string of biochemical reactions, including water evaporation, cell membrane rupture, protein coagulation, denaturation, and cell death, ultimately leading to the necrosis and death of the tumor tissue. The technique involves inducing thermal damage and high temperatures to cause tumor cell destruction. Elevated temperatures can damage cell membranes, denature, and coagulate cells. In addition, elevated temperatures can impact cell nuclei and other cell structures, causing DNA damage and cell death. Additionally, LITT has proven effective in completely eradicating glioma cells with an adequate thermal dose.^[^ ^87^ ^]^ However, challenges remain in achieving uniform thermal delivery to tumor regions, as observed in trials involving nanoparticles like gold–silica nanoshells, which face issues of heterogeneous distribution when administered intravenously.^[^ ^88^ ^]^ This technology's development marks a notable advancement in brain tumor treatment, offering improved safety and efficacy.

LITT, initially challenged by the high heat causing increased intracranial pressure during brain tumor treatments, has evolved significantly. Innovations include integration with MRI thermometry for real‐time temperature monitoring and engineered cooling systems.^[^ ^89^ ^]^ These advancements enable targeted necrosis in brain tissues at specific temperatures and durations. LITT's efficacy has been demonstrated in both animal models and human trials, showing a safer profile and reduced neurological morbidity compared to traditional surgical resection.^[^ ^90^ ^]^ In a study by Mohammadi et al., 34 patients with difficult‐to‐access high‐grade gliomas (DTA‐HGGs) underwent LITT using the NeuroBlate system.^[^ ^91^ ^]^ The study, which included 24 glioblastoma and ten anaplastic cases, assessed the extent of thermal damage using thermal damage threshold (TDT) lines (43°C for 2 or 10 min). Post‐treatment follow‐up revealed a median progression‐free survival (PFS) of 5.1 months, with patients with more comprehensive tumor coverage by TDT lines showing better outcomes. The study demonstrates LITT's efficacy in treating DTA‐HGGs, correlating greater tumor coverage with improved PFS, analogous to the extent of surgical resection. Similarly, Misios et al. found that the broader application of thermal ablation resulted in increased rates of progression‐free survival.^[^ ^92^ ^]^ Shawn outlined the potential benefits of LITT, including decreased blood loss, shortened hospital stays, smaller incisions, and faster wound healing.^[^ ^93^ ^]^ Missios et al. analyzed the evolution of LITT as a neurosurgical laser tool, encompassing its development, usage, indications, and effectiveness concerning neurosurgical applications (Figure 10C).^[^ ^89^ ^]^ Despite significant advancements in overcoming the initial technical challenges of LITT, the method is limited in its capacity to treat bigger or irregular lesions, as well as in coping with neurogliomas that are located near major vascular structures and the occurrence of postoperative edema. At present, there is inadequate data regarding the long‐term survival rates and quality‐of‐life outcomes of patients affected by high‐grade glioma upon undergoing LITT therapy.

### 3.3 Cryosurgery

**FIGURE 11:**
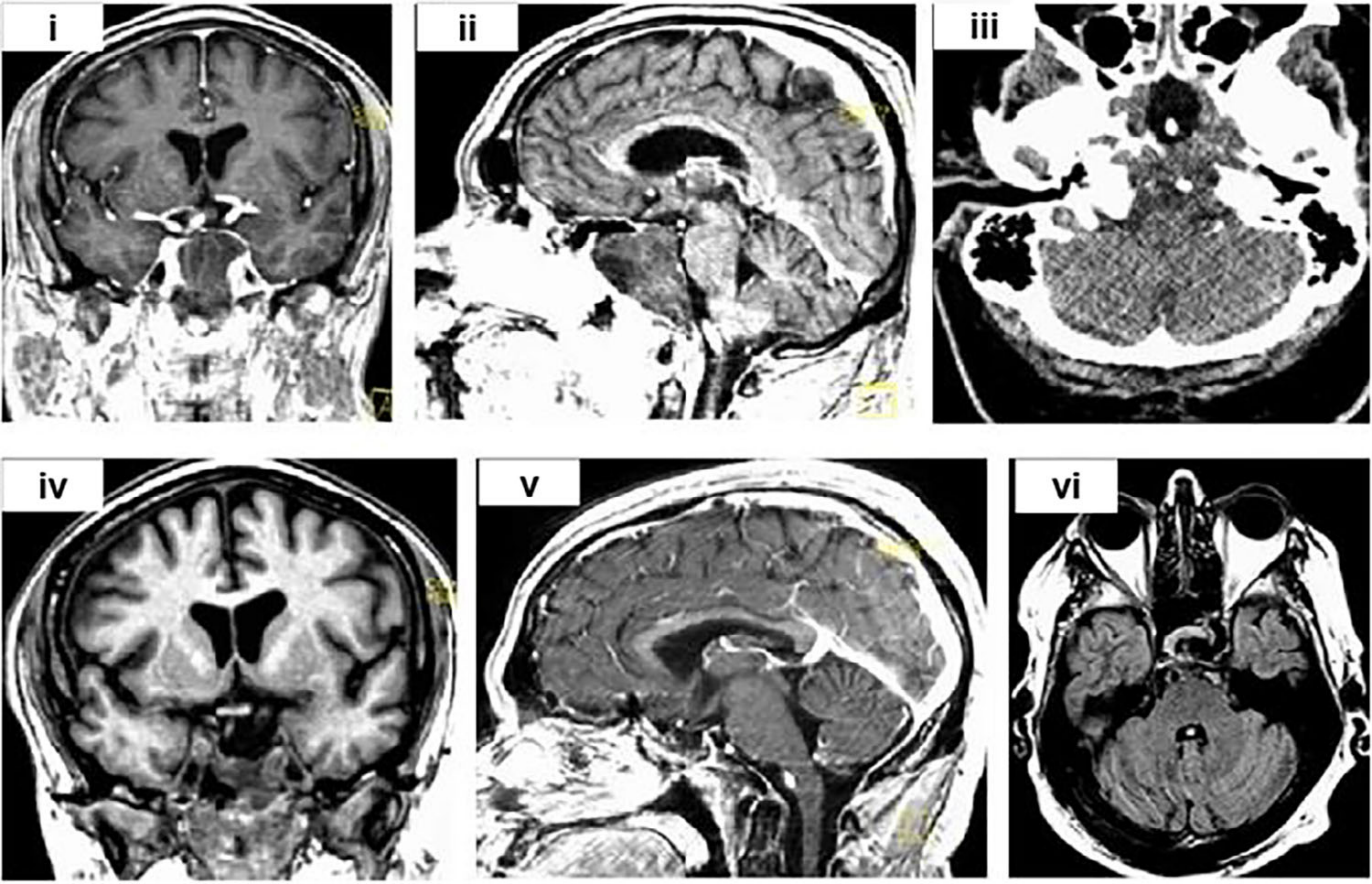
Cryosurgery for brain tumors. MRI images of a large pituitary tumor (i,ii). CT image with the formation of ice balls (shown as black voids) around the probe in the tumor (iii). Coronal, sagittal, and axial views of the MRI, with a significant reduction in tumor size (iv,vi). Reproduced under the terms of the CC‐BY 4.0 license.^[^ ^94^ ^]^ Copyright 2021, The Authors, published by Springer Nature.

Cryosurgery is a minimally invasive procedure for treating brain tumors by destroying cancerous tissue through the application of extreme cold. The underlying biophysical principle of cryosurgery is that cells exposed to low temperatures experience necrosis and resulting death. Cryosurgery uses the extreme cold produced by liquid nitrogen or argon gas to destroy cancer cells. The procedure involves inserting a small probe through an opening in the skull and guiding it to the tumor.^[^ ^94^ ^]^ The probe is cooled to a temperature ranging from −80 to −196°C with either liquid nitrogen or argon gas, which results in the formation of ice crystals inside the tumor cells. This process induces damage to the cell membrane and cell wall to some extent, thereby affecting normal cells’ function. Furthermore, the temperature drops result in water molecules crystallizing in the tissue, leading to the formation of ice crystals. These ice crystals can severely harm both the cells and adjacent tissues. The treatment leads to cell death and tumor shrinkage or disappearance. Cryosurgery provides numerous benefits over conventional brain tumor treatments, such as reducing the risk of harming healthy brain tissue, decreasing the risk of infection, and hastening recovery time. Recent research indicates that cryosurgery may effectively treat recurrent GBM and other brain tumor types.^[^ ^95^ ^]^ As shown in Figure 11, a patient with a large pituitary tumor was treated with cryosurgery following prior surgery and radiation therapy and subsequently experienced a significant reduction in tumor size without any complications.^[^ ^94^ ^]^ The cryosurgical procedure was aided by imaging equipment such as a CT or MRI, allowing the doctor to accurately locate and monitor the position of the cooling needle and surrounding tissues. Real‐time imaging and monitoring permit physicians to accurately determine the freezing range, ultimately enhancing the success rate of the treatment. Tacke et al. found that MRI‐guided cryosurgery using cryoprobes provided a precision prediction of cell necrosis produced by cryotherapy.^[^ ^96^ ^]^ Additionally, Zhang et al. reported that stereotactic‐guided cryosurgery successfully removed brain tumors and obliterated any residual tumors in the region.^[^ ^97^ ^]^ In a study involving 71 patients, cryosurgery facilitated tumor removal in 64 cases and was used to destroy residual neoplasms in seven cases where removal was incomplete. The use of intraoperative real‐time ultrasonic imaging enabled precise tumor delimitation and monitoring during cryosurgery, enhancing visualization in the central nervous system.^[^ ^98^ ^]^

While cryosurgery carries a potential risk of severe systemic reactions (cold shock), it is relatively minor compared to more advanced techniques. Additionally, there is a higher likelihood of bleeding complications due to the absence of clotting during freezing.^[^ ^99^ ^]^ Therefore, cryosurgery is limited in its application in the treatment of brain tumors. Tumors located in critical areas of the brain cannot undergo cold ablation to avoid functional damage. The complexity of brain tissue heightens the difficulty and risks of the surgery, requiring precise regulation of temperature and freezing range to prevent harm to healthy brain tissue. Cryosurgery may not completely eradicate brain cancer, necessitating the combination of other treatment techniques to augment curative outcomes. Therefore, a thorough contemplation of the patient's entire state is imperative when opting for a treatment plan.

Hyperthermia and cryotherapy are both widely used methods in tumor treatment, each with its own distinct advantages and specific applications. Hyperthermia destroys tumor cells, induces cell apoptosis, activates the immune response, and enhances the body's ability to combat tumors. Additionally, hyperthermia promotes drug penetration and absorption by melting or necrotizing tumor tissue. Cryotherapy, on the other hand, locally freezes and destroys tumor cells, impeding their proliferation and spread while also minimizing harm to adjacent healthy tissues. This fast procedure can be carried out under local anesthesia, thus making it ideal for localized treatments. The choice between hyperthermia and cryotherapy depends on factors such as tumor type and location, preoperative evaluation, treatment objectives, and the patient's overall health. In general, hyperthermia therapy is typically utilized for larger brain tumors, although it may cause additional harm to surrounding nerves and tissues. This treatment is usually recommended for situations where the brain tumor is challenging to operate on or if symptom relief is required. Alternatively, cryotherapy is better adapted for smaller brain tumors, though it may have limited effectiveness for larger ones. Cryotherapy is frequently prescribed for small brain tumors that are comparatively easier to remove.

## 4 PHOTODYNAMIC THERAPY (PDT) FOR BRAIN TUMORS

Photodynamic therapy (PDT) is a medical procedure that uses optical diagnosis and treatment and is commonly employed to treat various conditions, including cancer, skin disorders, and eye diseases. In particular, PDT is widely used in the treatment of brain tumors to manage residual tumors after surgery and prevent postoperative recurrence. Utilizing a photosensitizer (PS) and specific wavelengths of light, PDT can destroy tumor cells directly and achieve therapeutic effects by generating reactive oxygen species and modifying the tumor microenvironment. This treatment effectively controls residual tumors while minimizing invasiveness and side effects. The PS, which can generate reactive oxygen species under specific light wavelengths, is injected into the patient's body and delivered to the tumor tissue through the bloodstream during PDT.^[^ ^100^ ^]^ PDT can cause photosensitivity reactions, which require patients to avoid exposure to strong light sources. However, these side effects are often manageable, and PDT generally has a favorable impact on quality of life due to its minimally invasive nature. To maximize penetration depth and minimize light dispersion in tissue while optimizing PS activation, the wavelength range has been set between 600 and 850 nm. Shorter wavelengths can lead to skin photosensitivity, whereas longer wavelengths may not possess sufficient energy to trigger PS.^[^ ^101^ ^]^

### 4.1 PDT mechanism

**FIGURE 12:**
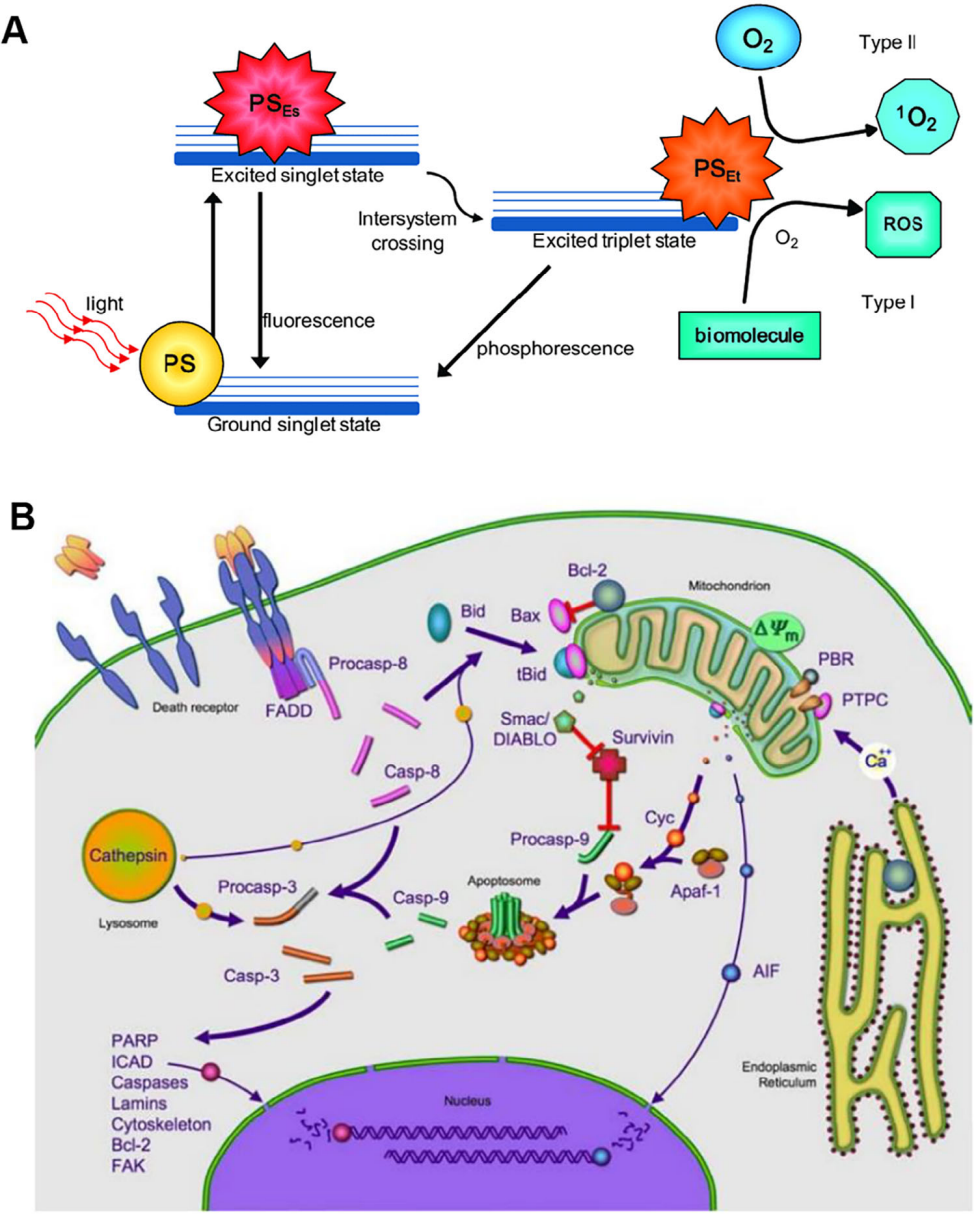
The mechanism of PDT for brain tumors. (A) Mechanism of PDT reaction. Reproduced under the terms of the CC‐BY 4.0 license.^[^ ^108^ ^]^ Copyright 2016, The Authors, published by MDPI. (B) Apoptosis mechanism related to PDT. Reproduced under the terms of the CC‐BY 4.0 license.^[^ ^109b^ ^]^ Copyright 2009, The Authors, published by IMR Press.

The mechanism of action for PDT involves three main steps: activating the PS, generating reactive oxygen species, and destroying tumor cells.^[^ ^102^ ^]^ When the PS within the tumor tissue is exposed to specific light wavelengths, it absorbs energy and undergoes photochemical reactions to produce reactive oxygen species. This produces oxidative stress in the tumor cells, damaging their membranes and ultimately causing cell death and injury. Reactive oxygen species can induce changes in the tumor microenvironment, stimulate immune responses, disrupt tumor blood vessels, and further inhibit tumor growth and metastasis. There is evidence indicating that PS can selectively accumulate in the tumor.^[^ ^103^ ^]^ It may interact with the tumor via the low‐density lipoprotein (LDL) receptor.^[^ ^104^ ^]^ Since LDL receptor levels are elevated in cancer cells, malignant cells preferentially uptake the LDL‐PS complex.^[^ ^105^ ^]^ Furthermore, high levels of PS are present in tumor‐associated macrophages within these regions.^[^ ^106^ ^]^ The selective uptake of PS by tumor cells may be due to various factors, including lower intracellular pH, microvascular leakage, impaired tumor lymphatic drainage, and increased collagen content.^[^ ^107^ ^]^ As shown in Figure 12A, upon entering the cells, the PS is irradiated with light at specific wavelengths that match its absorption spectrum, prompting the transition from the ground state (S0) to the first excited state (S1) as a result of photon absorption.^[^ ^108^ ^]^ Some of the energy is emitted as fluorescent light, and the remaining energy guides the PS molecules into the triplet excited state (T1), which is the preferred form for effective treatment.^[^ ^101^, ^106^ ^]^ When exposed to specific wavelengths, the PS generates singlet oxygen, which reacts with the tumor and eradicates it.

Tumor cell destruction can occur through various mechanisms, including direct cell damage due to apoptosis or necrosis, as well as indirect induction of inflammation and an immune response targeting the tumor microenvironment and vascular system.^[^ ^109^ ^]^ Apoptosis is a precise mechanism for regulating cellular self‐destruction, playing a crucial role in maintaining normal tissue structure, development, and cancer treatment. PDT may be the most rapid means of inducing cell apoptosis, given a well‐designed light activation protocol.^[^ ^110^ ^]^ Figure 12B illustrates the pathways through which cancer cell apoptosis is activated, including the extrinsic pathway and the intrinsic pathway.^[^ ^109b^ ^]^ The intrinsic pathway, primarily centered around mitochondria, serves as the predominant activation mechanism for PDT‐induced cell apoptosis, with the Bcl‐2 protein family playing a significant role in regulating apoptosis at the mitochondrial level. These two pathways converge at critical steps involved in caspase activation. When PDT induces apoptosis and necrosis in tumor cells, it releases tumor‐associated antigens (TAAs). These TAAs get taken up and processed by antigen‐presenting cells (APCs), triggering an immune response from CD8^+^ T cells (CTLs) and CD4^+^ T cells (helper T cells), which further induce anti‐tumor immunity. Kabingu et al. have demonstrated that PDT reduces tumors at primary and secondary sites by increasing the infiltration of CD8^+^ T cells.^[^ ^111^ ^]^ Another mechanism of action involves the alteration of the tumor microenvironment by PDT. The application of PDT results in the demise of tumor cells and the discharge of signaling molecules and cytokines, including tumor necrosis factor‐alpha (TNF‐α) and interleukin‐6 (IL‐6), leading to inflammation.^[^ ^112^ ^]^ The increase in inflammatory mediators can activate immune cells, and amplify the immune response, promoting the proliferation and activation of T cells. PDT has displayed immunomodulatory potential by inducing inflammatory reactions. Gollnick et al. have explored the role of cytokines in PDT‐induced local and systemic inflammation.^[^ ^113^ ^]^ It is found that PDT induces neutrophil migration into treated tumors, correlated with a transient surge in the expression of chemokines, IL‐6, and adhesion molecules. Additionally, PDT can lead to the destruction of tumor blood vessels and reduce the blood supply to the tumor. PS remaining in circulation after light excitation can cause vascular damage through the endocytic pathway mediated by the LDL receptor, leading to thrombosis and microvascular occlusion.^[^ ^114^ ^]^ This condition can cause tumor hypoxia and nutrient deprivation, making tumor cells more susceptible to immune cell attack. Notably, while dyes exhibit greater affinity for tumor tissue, they can also induce skin photosensitivity. Patients must, therefore, limit exposure to sunlight on the eyes and skin for thirty days or more, depending on the PS employed.^[^ ^115^ ^]^

### 4.2 Photosensitizer (PS)

**FIGURE 13:**
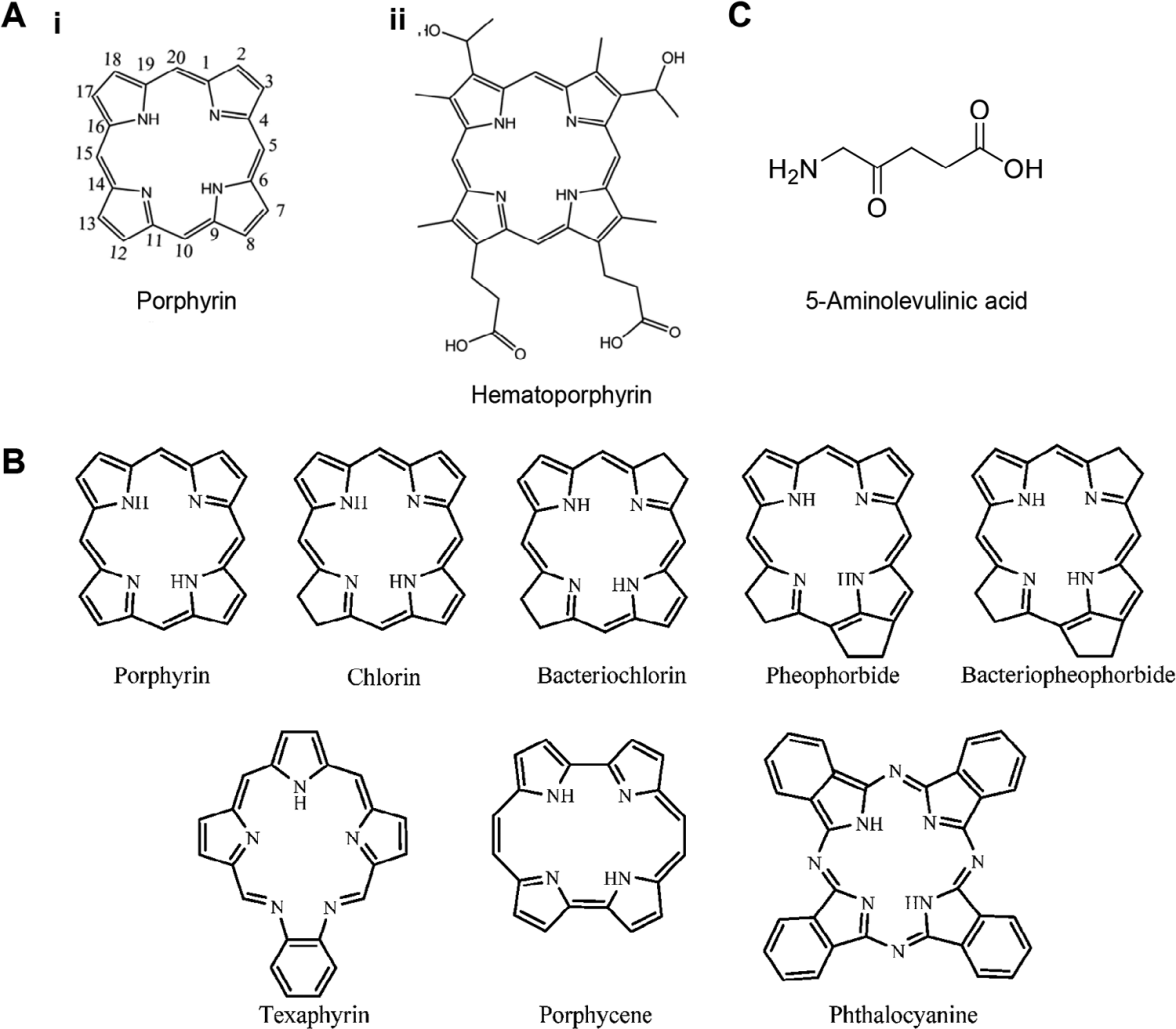
The structure of photosensitizers. (A) Basic structure of hematoporphyrin and porphyrin photosensitizers. Reproduced with permission.^[^ ^119^ ^]^ Copyright 2020, Royal Society of Chemistry. (B) Basic structure of porphyrinoid photosensitizers. Reproduced under the terms of the CC‐BY 3.0 license.^[^ ^116^ ^]^ Copyright 2013, The Authors, published by MDPI. (C) Molecular formula of a 5‐aminolevulinic acid molecule. Reproduced under the terms of the CC BY‐NC 4.0 license.^[^ ^131^ ^]^ Copyright 2018, The Authors, published by John Wiley & Sons, Inc.

PS has the potential to function as a diagnostic tool or adjunct during surgeries for tumor delineation, in addition to its use as an anti‐cancer therapeutic.^[^ ^116^ ^]^ PDT comprises three primary components, one of which is PS. The ideal PS should fulfill specified criteria, including systemic non‐toxicity, accumulation within diseased tissue, and activation at wavelengths capable of penetrating deep into brain tissue.^[^ ^117^ ^]^ Various PSs have been approved by the Food and Drug Administration (FDA) for the treatment of malignant tumors, as shown in Table 2, including breast cancer, bladder cancer, and esophageal cancer.^[^ ^118^ ^]^ PS can be categorized as porphyrins or non‐porphyrins. Porphyrins are organic molecules found abundantly in nature and biological systems. As illustrated in Figure 13A, they consist of four pyrrole rings and four nitrogen atoms, forming a large planar ring structure.^[^ ^119^ ^]^ Porphyrin‐like compounds have been extensively utilized in the medical field for the diagnosis and treatment of various diseases, such as cancer, cardiovascular diseases, and autoimmune disorders.^[^ ^120^ ^]^ Dye sensitizers employed in PDT primarily comprise porphyrinoid compounds, including bacteriochlorins, chlorins, phthalocyanines (Pcs), and other related structures.^[^ ^121^ ^]^ These compounds possess an extended conjugated system and exhibit light absorption in the visible region, making them ideal‐colored compounds or dyes. Figure 13B illustrates the structures of these compounds.^[^ ^116^ ^]^

**TABLE 2:** Summary of approved PS for PDT in brain tumors.

PS	Chemical structure	Indications (Brain tumor types)	Clinical applications
Porfimer sodium	Porphyrin‐based	GBM, Astrocytoma	FDA‐approved for PDT
Temoporfin	Chlorin‐based	High‐grade gliomas	Used in Europe for PDT
Verteporfin	Benzoporphyrin derivative	Age‐related macular degeneration	Off‐label use in brain tumors
Aminolevulinic acid (ALA)	Precursor for Protoporphyrin IX	GBM, Brain metastases	Used for fluorescence‐guided surgery
Talaporfin	Chlorin‐based	Superficial and early‐stage tumors	Approved in Japan for PDT

The application of porphyrin‐based PS in PDT has been extensively investigated and can be categorized into three generations.^[^ ^119^, ^122^ ^]^ First‐generation PS molecules consist of naturally occurring porphyrins, which exhibit absorption at around 400 nm with limited excitation absorption at longer wavelengths.^[^ ^123^ ^]^ Hematoporphyrin derivatives (HpD) and Photofrin® are known as first‐generation PS, which have their maximal absorption at relatively short wavelengths (≈630 nm), low molar extinction coefficients, long half‐lives, and high skin accumulation associated with skin phototoxicity.^[^ ^124^ ^]^ The main drawbacks of first‐generation PS are their short absorption wavelength, poor water solubility, short circulation half‐life, low tumor selectivity, and skin phototoxicity.^[^ ^116^, ^125^ ^]^ Second‐generation PS has been introduced to overcome these limitations. These PS are chemically pure, have maximal absorption within the phototherapy window (600–850 nm), greater singlet oxygen formation rates, and higher molar extinction coefficients.^[^ ^126^ ^]^ The second‐generation PS compounds include porphyrin derivatives, 5‐aminolevulinic acid (5‐ALA), dihydroxyphenyl, and Pcs.^[^ ^127^ ^]^ Additionally, these PSs tend to aggregate, affecting their photochemical properties and the bioavailability of active sites. These PSs are typically lipophilic and insoluble in aqueous media, which greatly impedes their intravenous administration. To enhance its solubility in aqueous media, second‐generation PS has been incorporated into various nanocarriers, which has led to the formation of third‐generation PS, whether conjugated with active targeting agents or not.^[^ ^128^ ^]^ Third‐generation PS exhibits higher selectivity towards tumor cells, achieved through conjugation with modifiers, including nanoparticles (NPs) and antibodies (Abs).^[^ ^129^ ^]^ Their efficient design aims to minimize off‐target effects while optimizing pharmacokinetic properties and exciting absorption to maximize the effective PDT window while minimizing consequences.^[^ ^130^ ^]^ Over the past 5 years, the most extensively researched PS for GBM‐PDT include porphyrins, 5‐ALA, chlorins, and Pcs. Currently, 5‐ALA is the primary precursor used for the treatment of GBM (Figure 13C).^[^ ^131^ ^]^

PDT's reliance on light activation limits its use in deeper or less accessible brain tumors. Research into new photosensitizers with deeper tissue penetration and improved light delivery methods is needed to expand PDT's applicability. Recent studies have focused on developing PSs with non‐linear optical properties, enabling activation by near‐infrared light for deeper tissue penetration. Non‐linear optical PSs can be activated by two‐photon absorption, a process that permits the use of longer wavelengths, which are less harmful and more effective in penetrating biological tissues. Furthermore, the integration of PSs with NPs has emerged as a promising approach to enhance tumor selectivity and targeting. Nanoparticle‐conjugated PSs can exploit tumor‐specific cell surface receptors, delivering the PS directly to tumor cells and reducing off‐target effects.^[^ ^132^ ^]^ This development not only increases the precision of PDT but also minimizes the potential for skin photosensitivity and other side effects associated with PS activation. Additionally, ongoing research is exploring the synergistic effects of combining PDT with other treatment modalities, such as immunotherapy, to enhance the overall therapeutic outcome.^[^ ^133^ ^]^ The use of PSs in combination therapies opens new avenues for personalized and targeted cancer treatment strategies. These advancements in PS technology are critical for overcoming the current challenges in PDT for brain tumors, including limited light penetration and the need for selective targeting. The development of new PSs with improved optical and chemical properties represents a significant step forward in enhancing the efficacy and safety of PDT in brain cancer therapy.

### 4.3 PDT for brain tumors

**FIGURE 14:**
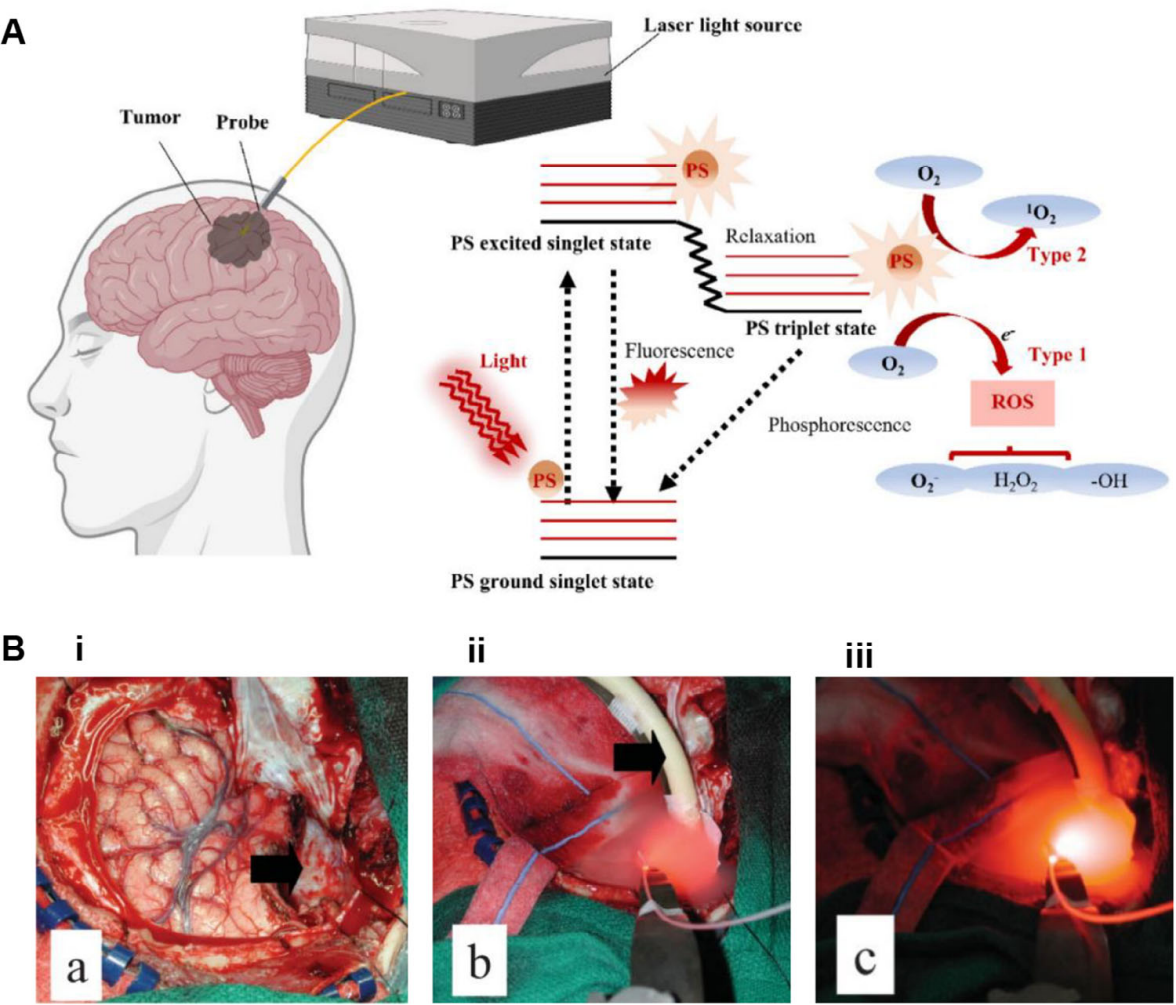
PDT for brain tumors. (A) Schematic diagram of PDT for GBM treatment and energy diagram of the oxygen‐dependent response. Reproduced under the terms of the CC‐BY 4.0 license.^[^ ^135^ ^]^ Copyright 2022, The Authors, published by MDPI. (B) Intra‐operative photographs: A temporal lobe tumor resection cavity (i); the cavity filled with 1:000 intralipid continuously irrigating the cavity via a hose (ii); laser on (iii). Reproduced with permission.^[^ ^140^ ^]^ Copyright 2006, Wiley Publishing.

PDT has been employed in pre‐ and post‐surgical resection to treat brain tumors. It can effectively control residual tumors and prevent postoperative recurrence, with over 80.0% of recurrences observed in the vicinity of the resection cavity.^[^ ^134^ ^]^ Performing PDT in the resection cavity is expected to significantly lower the risk of local recurrence. One technique used is interstitial PDT (iPDT), which involves the stereotactic insertion of optical fibers into the tumor mass.^[^ ^131^ ^]^ After administering photosensitizers to the patient, light stimulation is applied to the tumor, as depicted in Figure 14A.^[^ ^135^ ^]^ This minimally invasive approach aims to selectively destroy cancer cells. PSs are preferentially absorbed by cancer cells, resulting in higher accumulation levels compared to normal tissues. When exposed to specific light wavelengths, PSs are activated, producing reactive oxygen species (ROS) that can disrupt or destroy the cancer cells. Niels Finsen, a pioneer in the field of PDT, was awarded the Nobel Prize in 1903 for his discovery of using light therapy to treat skin tuberculosis.^[^ ^135^ ^]^ In 1978, T. J. Dougherty and his colleagues identified PDT as a potential anti‐cancer therapy for the treatment of skin or subcutaneous tumors, achieving complete or partial relief. Their successful application of PDT included treating breast cancer, colon cancer, and prostate metastases, marking a significant breakthrough in tumor treatment.^[^ ^136^ ^]^ Through further research on PDT delivery systems, biology, and mechanisms, the effectiveness of PDT in tumor therapy has been significantly enhanced.^[^ ^137^ ^]^ Studies suggest that the treatment's efficacy relies on the accumulation of photosensitizers in tumor tissues, the intensity of light irradiation, tissue penetration, and the availability of oxygen within cells.^[^ ^138^ ^]^ However, several studies have already proven the benefits of PDT not only in palliative care but also in the early stages and as an adjunctive therapy in surgery.

PDT has been extensively researched as an adjunctive therapy for brain tumors. Stummer et al. applied PDT on a patient with recurrent GBM who had undergone surgery, radiation, and chemotherapy. The patient ingested 5‐ALA and received laser irradiation, resulting in the tumor almost completely disappearing within ≈24 h, and showing no recurrence for 5 years thereafter.^[^ ^139^ ^]^ In 2006, Muller et al. treated 112 GBM patients, demonstrating a median survival time (MST) of 11 months in the PDT‐treated group compared to only 8 months in the control group (Figure 14B).^[^ ^140^ ^]^ Furthermore, Eljamel et al. treated GBM patients with PDT in 2010 and observed a survival increase of 1.5 years compared to the control group.^[^ ^141^ ^]^ By selectively targeting cancer cells and minimizing damage to healthy tissues, PDT has extended patients' survival. In a series of 365 PDT applications using 5‐ALA and sodium porphyrin on 150 brain cancer patients, only 4.7% experienced side effects, and 1.3% of recurrent tumor patients developed brain edema after sodium porphyrin‐mediated PDT.

In recent years, there has been a rising interest in utilizing the coupling of PS with NPs to enhance tumor selectivity and targeting while minimizing adverse effects.^[^ ^142^ ^]^ A promising approach is the development of nanoparticle‐conjugated PS that can exploit tumor‐specific cell surface receptors to directly deliver the PS to tumor cells.^[^ ^143^ ^]^ To address the limited penetration of light wavelengths through brain tissue required for optimal activation, researchers are developing NPs activated by near‐infrared light. This enables the release of photons at the necessary wavelength for PS excitation, allowing for deeper penetration into the tissue.^[^ ^144^ ^]^ The ultimate goal is to achieve enhanced tumor cell specificity, even in regions with an intact BBB, while also enabling the application of PDT at greater distances from the light source. This would permit the handling of significantly larger tissue volumes than presently feasible using PDT procedures. In general, PDT is a promising treatment option for certain types of brain tumors. PDT selectively targets cancer cells and minimizes damage to healthy tissue, thus improving patients' QOL and extending their survival. Ongoing research on new PSs and improved delivery methods may further optimize the effectiveness of PDT in the future. PDT stands out as a minimally invasive option that effectively controls residual tumors and prevents postoperative recurrence. Utilizing photosensitizers and specific light wavelengths, PDT targets tumor cells while minimizing invasiveness and side effects. Its application in brain tumor treatment highlights the continued innovation in therapeutic strategies.

## 5 TUMOR TREATING FIELDS (TTFIELDS) FOR BRAIN TUMORS

The tumor‐treating fields (TTFields) cancer treatment technique utilizes intermediate frequency (100–300 kHz) and low intensity (1–3 V cm^−1^) alternating electric fields. Non‐invasive application of these fields involves placing transducer arrays close to the skin‐tumor site. TTFields effectively inhibit tumor growth, and disrupt cell division, and ultimately destroy cancer cells. The electric field disrupts the division and growth of tumor cells by causing charge imbalances and interfering with the normal cell division process, ultimately leading to cell death. TTFields therapy is associated with minimal systemic side effects. The most common side effect is skin irritation under the device electrodes. TTFields have been shown to maintain or improve quality of life compared to standard chemotherapy alone.

### 5.1 Tumor treating fields (TTFields)

TTFields is a novel therapeutic technology used for treating brain tumors. It involves the application of a constant alternating electric field to the patient's head region via a device called Optune (also known as NovoTTF‐100A System). Optune consists of an electrode array that is attached to the scalp, a controller, and a power supply. The electrode array is designed with specific spacing and layout to ensure an even distribution of the electric field.^[^ ^145^ ^]^ The controller generates and regulates the electric field based on the prescribed frequency and intensity settings. The power supply provides the necessary energy for the device to function. However, the efficacy of TTFields can be limited by patient compliance and the variability in tumor response. It is important to note that patients must wear the Optune device for a minimum of 18 h per day, which has led to certain major adverse events associated with TTFields.^[^ ^146^ ^]^ Enhancing the comfort and convenience of the device, as well as personalizing treatment parameters, are crucial for improving patient outcomes.

**FIGURE 15:**
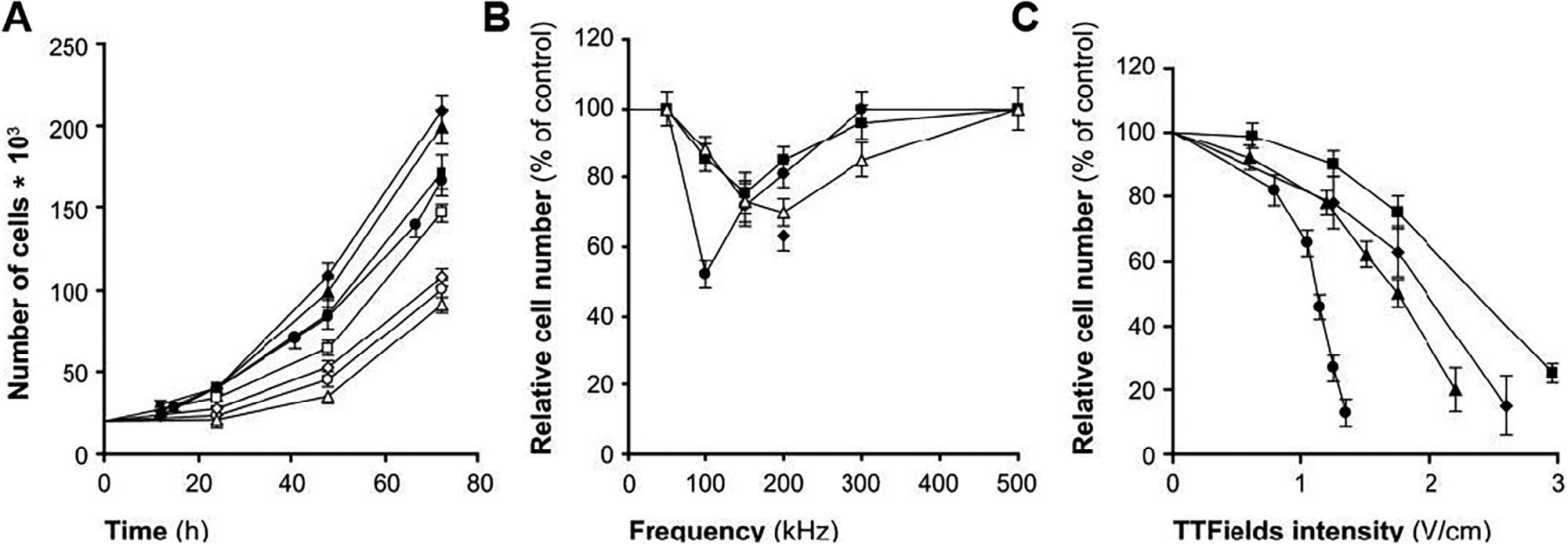
The impact of TTFields' frequency and intensity on the proliferation of various cancer cells was investigated. (A) Cell counts in untreated groups (represented by filled symbols) were compared to those in groups exposed to TTFields (represented by open symbols) over 24 h. (B) The relative change in cell count after 24 h of treatment with different frequencies of TTFields was analyzed. (C) The relative change in cell count after 24 h of treatment with varying intensities of TTFields was examined. The cancer cell lines were represented by different symbols: B16F1 cells were depicted by circles, MDA‐MB‐231 cells by squares, F‐98 cells by triangles, and H1299 cells by diamond‐shaped squares. Reproduced with permission.^[^ ^150^ ^]^ Copyright 2007, National Academy of Sciences, USA.

Some studies have found that the frequency of the alternating fields can yield different biological effects. Electric fields below 1 kHz (low‐frequency) impact cell membranes, altering their polarization states, and influencing the behavior of excitable tissues. This type of electric field modulates cell electrical activity by affecting the ion channels' activity, such as the action potential discharges in neuronal cells.^[^ ^147^ ^]^ Initially, intermediate frequency electric fields (100–500 kHz) were considered to be without any beneficial effects. The rapid alternation speed was deemed incapable of triggering action potential discharges and causing noteworthy tissue heating.^[^ ^148^ ^]^ However, recent research suggests that intermediate‐frequency electric fields may impact various biological processes, such as cell morphology, proliferation, and differentiation, by modulating changes in cell channels, ion channels, and protein activity.^[^ ^149^ ^]^ On the other hand, high‐frequency electric fields (above 500 kHz), known as radiofrequency electric fields, produce biological effects through dielectric and thermal mechanisms.^[^ ^25^ ^]^ The dielectric effect alters ion channels on cell membranes, while the thermal effect causes tissue heating and is employed in medical applications such as radiofrequency ablation therapy. Figure 15 demonstrates the influence of TTFields frequency and intensity on the proliferation of different cancer cells. Notably, for F‐98 rat glioma, the most effective frequency is observed at 200 kHz.^[^ ^150^ ^]^ Based on these findings, Eilon et al. documented the first successful use of TTFields in combating cancer in both in vitro and in vivo settings in 2004, which subsequently facilitated the completion of clinical trials of TTFields in GBM treatment.

### 5.2 TTFields mechanism

**FIGURE 16:**
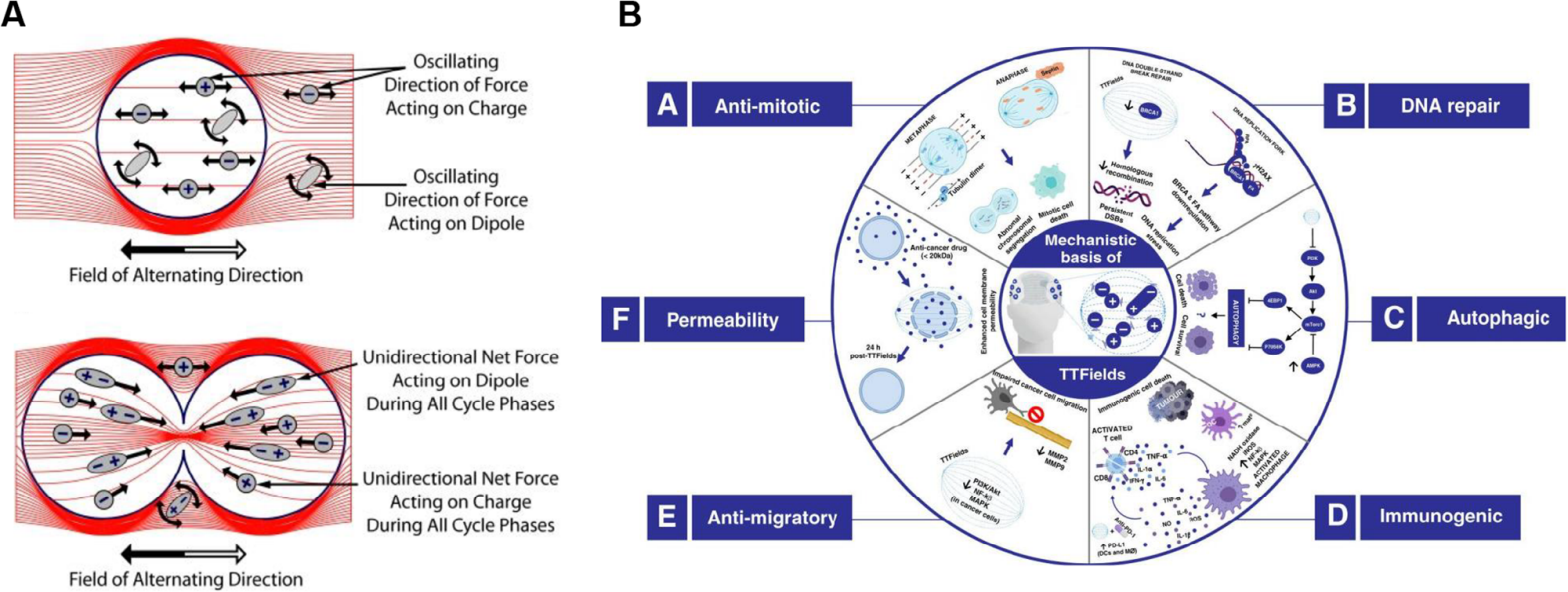
The mechanism of TTFields. (A) Distribution of AC field in both resting and dividing cells. Reproduced with permission.^[^ ^150^ ^]^ Copyright 2007, National Academy of Sciences, USA. (B) TTFields not only exert anti‐mitotic effects but also disrupt various biological processes, such as DNA repair, autophagy, cell migration, permeability, and immune response, leading to the induction of anti‐cancer effects. Reproduced under the terms of the CC‐BY 4.0 license.^[^ ^25^ ^]^ Copyright 2021, The Authors, published by Springer Nature.

Research on the mechanism of TTFields and its clinical potential to disrupt cellular polarity includes investigating their effects on the distribution of the tumor cell microenvironment and the movement of cancer cells. TTFields have been proven to inhibit the proliferation of cancer cells by disrupting the mitotic apparatus. Within an alternating current (AC) electric field, all charged particles and polar molecules experience forces that switch directions, causing ionic currents and dipole rotations to oscillate (Figure 16A). Bidirectional forces are applied to highly polarized intracellular components, such as microtubule proteins and septin molecules, inducing anti‐mitotic effects. This leads to abnormal microtubule aggregation and furrow formation during spindle formation, ultimately resulting in abnormal chromosome separation or cell death. Voloshin et al. have provided evidence that TTFields can induce anti‐mitotic effects on cancer cells by applying bidirectional forces to intracellular components, such as microtubule proteins and septin molecules.^[^ ^151^ ^]^ This leads to abnormal aggregation of microtubules and the formation of atypical furrows during cell division. Importantly, studies have shown that TTFields can effectively inhibit the migratory properties of cancer cells. In a comprehensive review, Wenger and colleagues discussed the computational methods used to characterize TTFields and summarized research on their macroscopic distribution in the human head as well as their microenvironmental distribution within tumor cells. Furthermore, researchers have investigated the impact of TTFields on cancer cell mobility and microenvironmental factor distribution within tumor cells to elucidate the mechanisms by which TTFields disrupt cellular polarity and explore their potential clinical importance.^[^ ^152^ ^]^

Although TTFields were initially shown to inhibit cancer cell proliferation by interfering with mitotic devices, increasing evidence supports the efficacy of TTFields through multiple intracellular mechanisms including the inhibition of cell proliferation and the disruption of biological processes such as DNA repair, autophagy, cell migration, permeability, and immune response, leading to anti‐cancer effects (Figure 16B).^[^ ^153^ ^]^ During TTFields treatment, the application of low‐intensity, alternating electric fields can downregulate the expression of genes involved in the BRCA and Fanconi anemia (FA) pathways. Mutations or defects in BRCA genes lead to a decrease in the efficiency of homologous recombination repair (HRR), while mutations in FA genes interfere with and disrupt the repair of double‐strand breaks (DSBs). Consequently, these mechanisms contribute to a reduction in DNA repair processes, increase tumor cells' sensitivity to DNA damage, and ultimately lead to apoptosis, cell cycle arrest, or enhanced sensitivity to other treatment approaches. When the PI3K/Akt/mTORC1 signaling pathway is activated, cells typically inhibit autophagy. However, TTFields treatment may disrupt the PI3K/Akt/mTORC1 signaling pathway, preventing it from suppressing autophagy. In addition, TTField treatment has been demonstrated to reduce cancer cell migration and invasiveness via modulation of multiple signaling pathways. Key signaling pathways involved include the nuclear factor (NF)‐κB, mitogen‐activated protein kinase (MAPK), and phosphatidylinositol3‐kinase (PI3K)/Akt pathways. TTField treatment enhances cell membrane permeability through tight junction creation, lipid bilayer insertion, or expansion of existing membrane channels. By increasing the permeability of the cell membrane, TTFields therapy potentially amplifies the sensitivity of cells to chemotherapy drugs. Furthermore, TTFields therapy enhances tumor immunity by influencing the activity of macrophages, leading to alterations in reactive oxygen species, nitric oxide, and pro‐inflammatory cytokine secretion. Additionally, it promotes immunogenic cell death and facilitates the recruitment and maturation of dendritic cells (DCs). Based on clinical trial results, TTFields therapy has been approved by the US Food and Drug Administration for the treatment of tumors such as GBM.^[^ ^154^ ^]^ Further research integration with clinical practice will advance comprehension of the interplay and potential applications of TTFields therapy in the realm of immunotherapy.

### 5.3 TTFields treat brain tumors

**FIGURE 17:**
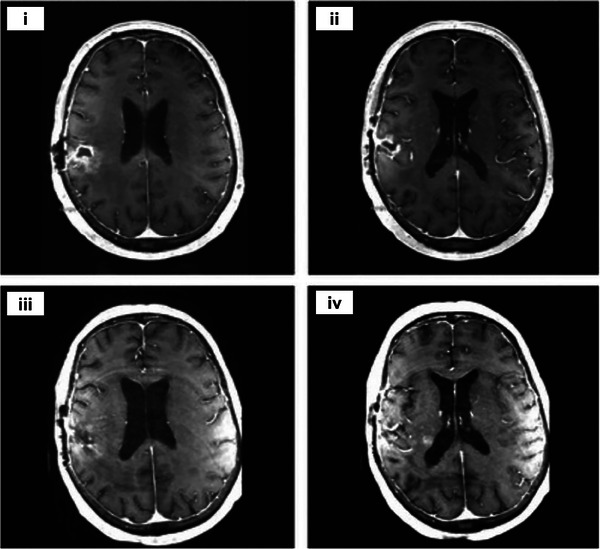
TTFields therapy against recurrent glioblastoma. The gadolinium‐enhanced T1‐weighted MP RAGE slices in the upper (i) and lower (ii) regions unambiguously exhibit the neoplasm. Following the administration of bevacizumab and TTFields, the original tumor remained stable (iii), whereas a novel pathological site (iv) was identified along the peripheral edge of the right lateral ventricle after 24 months. Reproduced under the terms of the CC‐BY 4.0 license.^[^ ^158^ ^]^ Copyright 2015, The Authors, published by John Wiley & Sons, Inc.

Adding TTFields to the standard treatment has been proven to prolong the survival of patients with newly diagnosed GBM, recurrent GBM, and brain metastases. The effectiveness of TTFields therapy is influenced by factors such as treatment duration, field intensity, and field frequency.^[^ ^160^ ^]^ The optimal frequency varies depending on the type of cancer, and for GBM, the recommended frequency for clinical use is 200 kHz.^[^ ^149^ ^]^ TTFields can be used as a monotherapy or in combination with other techniques, such as chemotherapy and radiotherapy, to improve prognosis.^[^ ^155^ ^]^ The EF‐14 trial was a randomized, double‐blind, placebo‐controlled research that was performed on patients who had newly diagnosed GBM. The primary aim was to investigate the impact of adding the chemotherapy drug temozolomide (TMZ) to the standard treatment (post‐surgical radiation and adjuvant chemotherapy) on overall survival.^[^ ^155^, ^156^ ^]^ The trial revealed that augmenting TMZ maintenance with TTFields considerably extended patients’ overall survival and decreased the likelihood of disease progression, highlighting it as a critical treatment strategy.^[^ ^157^ ^]^ In Figure 17, TTFields therapy was employed to manage recurrent glioblastoma.^[^ ^158^ ^]^ TTFields treatment was administered on the shaved skin of the patient by the NovoTTF‐100A system (FDA approved, Novocure, Inc., Haifa, Israel) at a frequency of 200 kHz. The gadolinium‐enhanced T1‐weighted MP RAGE slices in the upper (I) and lower (II) regions unambiguously exhibit the neoplasm. Following the administration of bevacizumab and TTFields, the original tumor remained stable (III), whereas a novel pathological site (IV) was identified along the peripheral edge of the right lateral ventricle after 24 months. Overall, TTFields represent a promising new modality for the treatment of brain tumors, and ongoing research is expected to further clarify their potential benefits.

Currently, several clinical trials are evaluating the efficacy of TTFields therapy in the treatment of brain metastases and other extracranial tumors. Particularly noteworthy is the STELLAR trial conducted in 2019, which examined the feasibility of combining TTFields with chemotherapy in patients with malignant pleural mesothelioma.^[^ ^159^ ^]^ The results were promising, showing a significant improvement in median overall survival (18.2 months) and median progression‐free survival (7.6 months) in contrast to the historical control group that only received chemotherapy (12.1 months and 5.7 months, respectively).^[^ ^160^ ^]^ In addition, TTFields therapy has obtained FDA approval for use in combination with chemotherapy for the treatment of malignant pleural mesothelioma. TTFields therapy is expanding its scope of application and demonstrating immense potential across various fields.

## 6 COLD ATMOSPHERIC PLASMA (CAP) FOR BRAIN TUMORS

Cold atmospheric plasma (CAP) is an emerging tumor treatment technology that utilizes highly reactive gas ions generated at ambient temperature and pressure to selectively target cancer cells. CAP induces a series of biochemical reactions and oxidative stress upon contact with cancer cells, leading to damage to intracellular organelles and the induction of apoptosis.^[^ ^161^ ^]^ CAP offers advantages such as minimally invasive treatment, non‐ionizing radiation, and ease of use. However, further research is needed to comprehend the impact of CAP on different types of cancer and to optimize its therapeutic indications and protocols. CAP shows potential as a selective cancer cell‐killing approach and holds promise as a fresh alternative in the field of cancer treatment.

### 6.1 Cold atmospheric plasma (CAP)

**FIGURE 18:**
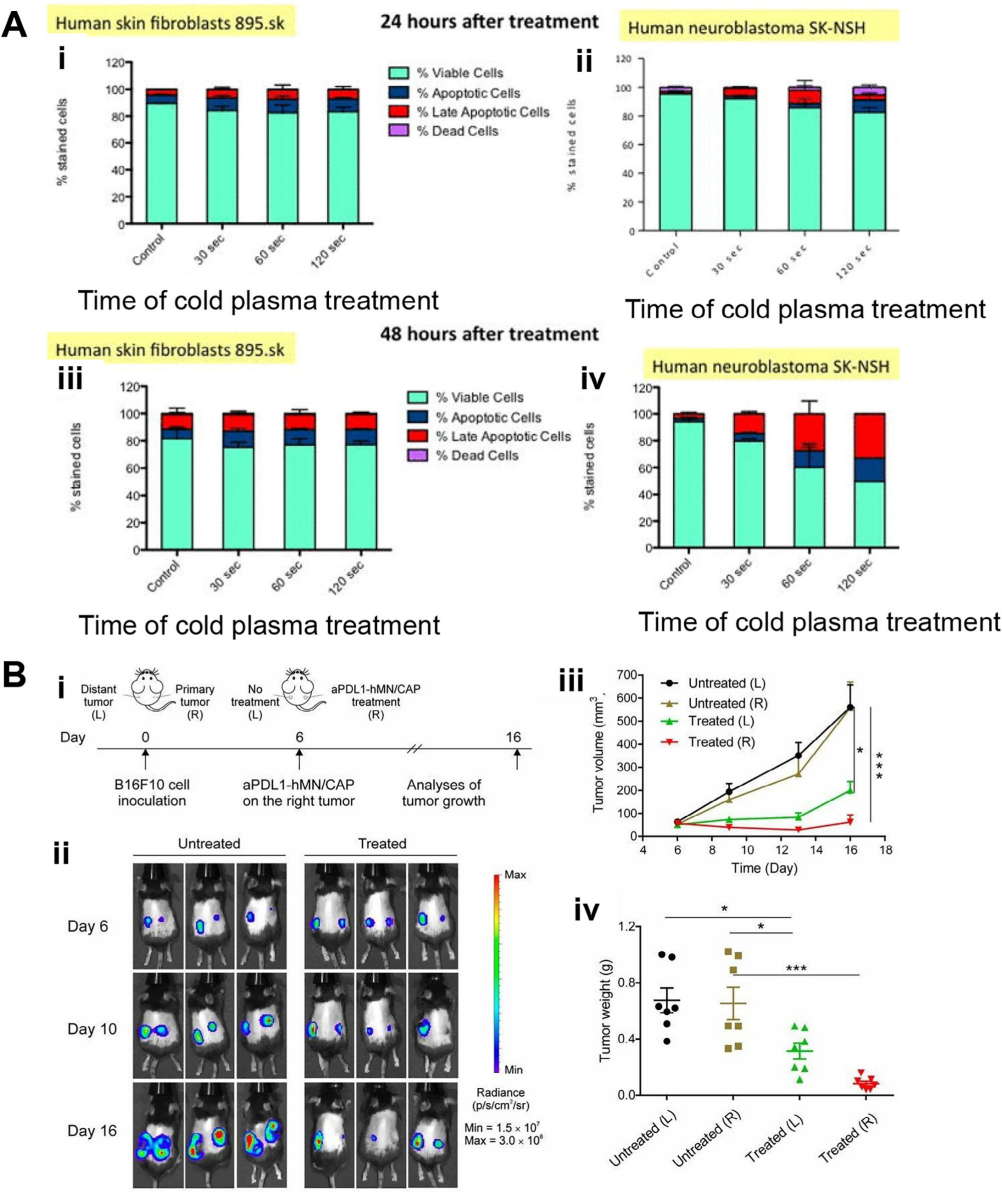
Anti‐tumor efficiency of CAP. (A) Selective killing effect of CAP treatment: cell survival of human skin fibroblasts treated with cold plasma of different durations after 24 and 48 h (i, ii). Cell survival of neuroblastoma treated with cold plasma of different durations after 24 and 48 h (iii, iv). Reproduced with permission.^[^ ^166^ ^]^ Copyright 2013, AIP Publishing. (B) Transdermal atmospheric pressure cold plasma combined with immune checkpoint inhibitor therapy. Schematic diagram, right tumor treated with CAP combined with immune checkpoint therapy, left tumor untreated (i). Mouse tumor bioluminescence images (ii). The curve of tumor volume (iii). Changes in tumor weight (iv). Reproduced with permission.^[^ ^172^ ^]^ Copyright 2020, National Academy of Sciences, USA.

As one of the fourth states of matter, plasma is a highly energetic and widespread form of matter, constituting up to 99% of the universe's matter. In 1857, Siemens invented the ozone generator, which utilized electronic discharge to produce ozone, thereby initiating the practical application of plasma technology. Over more than a century, plasma technology has been applied in numerous fields such as energy, materials, semiconductors, aerospace, metallurgy, biomedical, and agriculture.^[^ ^162^ ^]^ Depending on the gas temperature, plasma can be classified into high‐temperature plasma and low‐temperature plasma. High‐temperature plasma consists of gas that is nearly entirely ionized, with high temperatures for both heavy particles and electrons. Low‐temperature plasma comprises partially ionized or un‐ionized gas and can be categorized into thermal equilibrium plasma and non‐thermal equilibrium plasma. In thermal equilibrium plasma, the electron temperature equals the ion temperature, resulting in a high macroscopic temperature. Meanwhile, non‐thermal equilibrium plasma, also known as cold plasma, exhibits an electron temperature of about 1000 K but can have lower temperatures for heavy particles like ions and atoms, approaching room temperature. The macroscopic temperature of plasma is determined by the temperature of the heavy particles, therefore giving it the name cold plasma for non‐thermal equilibrium plasma, as it can be produced at atmospheric pressure. It is also commonly referred to as CAP. In the 1970s, the publication of plasma‐based sterilization marked the beginning of a new era in plasma biomedical research. The CAP technology has since undergone rapid development and received extensive attention, finding wide applications in diverse fields including biomedicine, environmental conservation, food safety and agriculture, catalysis, and material processing.^[^ ^163^ ^]^ The efficacy of CAP applications is chiefly attributed to the holistic impact of the produced ions, electrons, reactive oxygen species (ROS), reactive nitrogen species (RNS), and other physicochemical species.^[^ ^164^ ^]^ Fridman et al. presented the first analysis of CAP treatment's anti‐cancer properties in 2007, marking a significant breakthrough in medical research and the application of CAP technology.^[^ ^165^ ^]^ Subsequent studies by Keidar and his colleagues have demonstrated that CAP inhibits the growth of cancer cells across various cell lines while sparing normal cells (Figure 18A).^[^ ^166^ ^]^

Presently, two primary types of CAP tumor treatment devices are under development. One type of treatment involves the use of a plasma jet, like the device developed by Liu's team at Xi'an Jiaotong University, which utilizes plasma generated from helium gas under different polarity conditions to treat bone cancer cells.^[^ ^167^ ^]^ The other type utilized a dielectric barrier discharge (DBD) with a surface medium, such as the one used by Li et al. at Yonsei University in South Korea, for direct treatment of human glioma cells cultured in dishes.^[^ ^168^ ^]^ Other applications of CAP include plasma‐activated medium (PAM), nanoparticle synergy, and substance delivery.^[^ ^169^ ^]^ Utsumi et al. attempted to suppress tumor growth by injecting PAM into tumor tissue in mice.^[^ ^170^ ^]^ Furthermore, Professor Kim from South Korea provided an overview of the specific effects of certain NPs in the CAP treatment of tumor cells, highlighting the potential synergistic application of CAP and NPs in the medical field.^[^ ^171^ ^]^ In a study conducted by the research team led by Chen Zhitong, where the applicant participated, they employed millimeter‐scale hollow tubes to directly deliver plasma to subcutaneous tumor tissue. This approach, when used in conjunction with immune checkpoint inhibitors, resulted in a remarkable reduction in tumor growth. Interestingly, the untreated side of the tumor tissue also experienced inhibition due to the immune response triggered by CAP (Figure 18B).^[^ ^172^ ^]^

### 6.2 CAP mechanism

**FIGURE 19:**
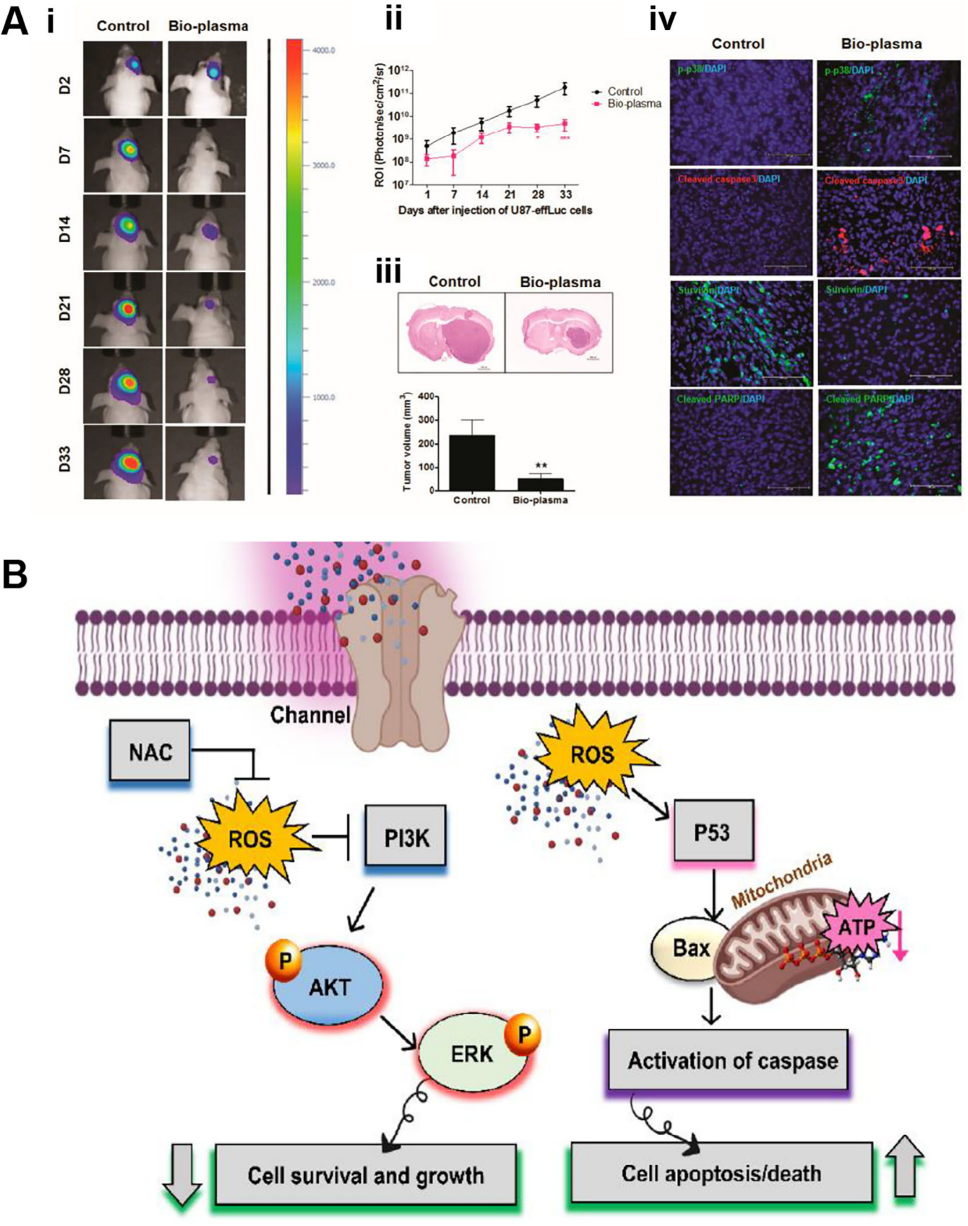
The mechanism of CAP against brain tumor. (A) The effects of CAP treatment on tumor growth. Bioluminescence imaging visualized changes in the tumor region of interest (i) after plasma treatment (ii). Tumor volume and size were measured using bioluminescence imaging in sectioned mouse brain tumors (iii). Immunofluorescence analysis demonstrated increased expressions of apoptotic proteins including cleaved caspase‐3, p‐p38, and cleaved PARP (iv). These findings suggest the induction of cell death in treated tumors. Nonthermal plasma exhibits potential for promoting tumor regression and stimulating apoptosis in brain tumor cells. Reproduced under the terms of the CC BY license.^[^ ^175^ ^]^ Copyright 2020, The Authors, published by MDPI. (B) Cellular mechanism of CAP in brain tumors. Reproduced under the terms of the CC BY license.^[^ ^78^ ^]^ Copyright 2022, The Authors, published by MDPI.

U87 MG, a highly sensitive brain cancer cell line that is extensively studied, is well‐known in the scientific community.^[^ ^173^ ^]^ Recent research has provided valuable insights into the significant role played by cold plasma, specifically in U87 MG cells. This effect is particularly evident through the activation of the mitogen‐activated protein kinase (MAPK) signaling pathway, which appears to remain unaffected in normal cells.^[^ ^174^ ^]^ The tangible outcomes of utilizing plasma treatment are demonstrated through a significantly elevated survival rate in the plasma‐treated group compared to the untreated group, along with remarkable reductions in tumor size in mouse models (as depicted in Figure 19A).^[^ ^175^ ^]^ These findings validate the presence of non‐thermal effects and emphasize the complexity associated with plasma exposure, necessitating a comprehensive evaluation of potential health implications for future research. Moreover, the elevated level of reactive oxygen and nitrogen species (RONS) within cancer cells following low‐temperature plasma treatment poses further obstacles linked to the oxidative damage induced by RONS found in the treated plasma. In contrast, healthy cells exhibit greater capability in protecting themselves against these consequences.

In CAP biomedical applications, ROS/RNS plays a pivotal role. Low concentrations of ROS/RNS promote cell growth, whereas high concentrations can trigger cell apoptosis via protein, DNA, and endoplasmic reticulum damage.^[^ ^176^ ^]^ It has been reported that cancer cells uptake ROS to induce apoptosis in brain tumors via intrinsic or extrinsic pathways. Cancer cells also exhibit distinct mechanistic features when compared to normal cells. Figure 19B illustrates the cellular mechanism of CAP in brain tumors.^[^ ^78^ ^]^ The PI3K signaling pathway, which is involved in promoting cell survival and growth, has been implicated in promoting apoptosis in the central nervous system. AKT or PKB serves as the primary protein effector downstream of this pathway. Furthermore, ERK plays a pivotal role in the growth mechanism by regulating the PI3K/AKT pathway. Reactive oxygen species (ROS) produced by nonthermal plasma promote the pathophysiology of apoptosis in brain tumor cells. Additionally, the effect of p53 on caspase regulation is intricately linked to the mitochondrial mechanism. Hence, Bax stimulates the release of cytochrome c and activates caspase signaling, leading to an upsurge in cell apoptosis and overall cell death.

### 6.3 CAP treat brain tumors

**FIGURE 20:**
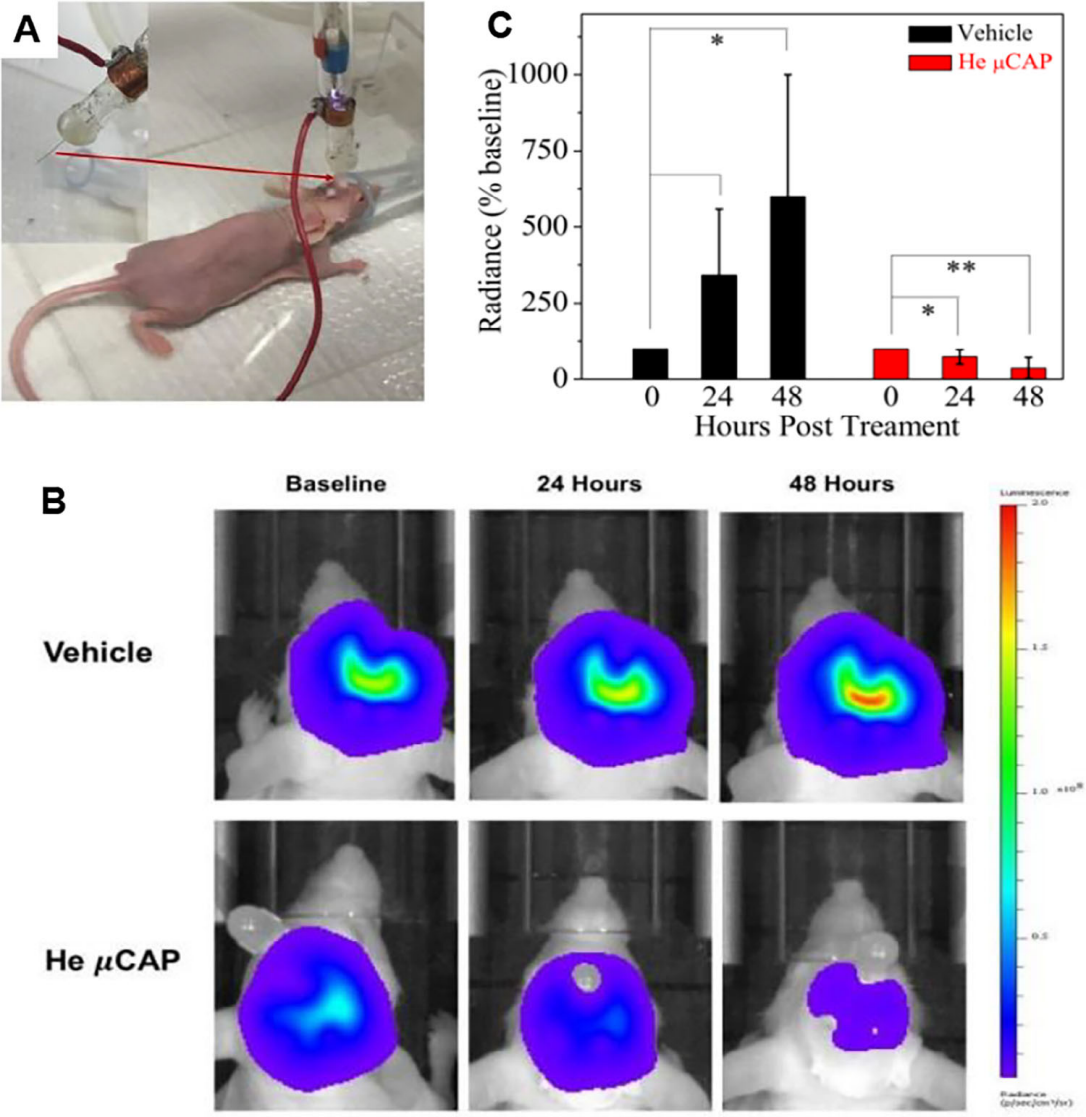
Targeting GBM tumors in vivo using µCAP. (A) micro‐sized CAP device imaging. (B) The size of the tumor in mouse brain slices using bioluminescent imaging and tumor volume. (C) CAP administration kept tumor volume below baseline compared to the control group. Reproduced under the terms of the CC BY license.^[^ ^178^ ^]^ Copyright 2017, The Authors, published by MDPI.

Numerous research results indicate that CAP has the potential for clinical cancer treatment by inhibiting tumor growth.^[^ ^175^, ^177^ ^]^ Furthermore, CAP can promote differentiation, inhibit angiogenesis, sensitize drugs, prevent migration, induce apoptosis, and inhibit proliferation. The impact of a micro‐sized CAP device (µCAP) on GBM was confirmed in Figure 20,^[^ ^178^ ^]^ with results demonstrating a significant reduction in tumor volume for CAP‐treated GBM cells compared to the control group. Chen et al. used a portable air‐fed CAP (a‐CAP) device to treat the post‐surgical tumors.^[^ ^179^ ^]^ They found that administering local a‐CAP treatment in the surgical cavity can effectively induce in situ death of cancer cells in residual tumor cells, without any adverse effects on normal cells. Moreover, it initiated a strong T‐cell‐mediated immune response to suppress residual tumor cells. Recent studies reveal CAP as a potent method for inducing apoptosis in brain cancer cells through the generation of RONS. Both in vivo and in vitro studies have highlighted the pivotal role of treatment duration and dosage of CAP in cancer suppression. Furthermore, treatment duration is a determining factor in the extent of cellular toxicity and apoptosis due to the cell cycle arrest that promotes tumor suppression. A significant amount of evidence indicates that plasma‐based therapies can effectively induce cell death in cancer cell lines by increasing morphological changes, inducing cell cycle arrest, and activating apoptosis genes.^[^ ^175^, ^180^ ^]^

Notably, plasma‐based therapies exhibit limited penetration depth, potentially impeding the delivery of bioactive agents. Researchers have thus explored novel strategies to improve the delivery efficiency of plasma‐generated reactive species. Chen et al. have developed a microneedle with a hollow structure specifically for plasma transdermal delivery, facilitating the entry of CAP into tumors through the skin.^[^ ^172^ ^]^ The research indicates that although the importation of ROS and RNS has diminished, they are still present and have notable effects. Moreover, CAP can not only kill cancer cells and inhibit tumor growth but also promote DC maturation, which initiates T‐cell‐mediated immune response. The outcomes of CAP treatment illuminate the anti‐cancer properties of CAP, indicating its potential to cure cancer in the future while sparing healthy tissues, which could potentially offer significant benefits to humanity in the future. As an emerging treatment, CAP therapy's side effects are not fully understood. However, early studies suggest that it is well‐tolerated with minimal adverse effects, potentially leading to a positive impact on quality of life, particularly for patients with limited treatment options.

While radiotherapy has been a traditional mainstay for brain tumor treatment, with median survival rates varying based on tumor type and grade, ablation therapies like MWA and laser interstitial thermal therapy have emerged as effective for localized tumors. However, ablation therapy typically demonstrates better outcomes in smaller, well‐defined tumors, whereas radiotherapy provides broader coverage beneficial in more diffuse or invasive tumors. PDT has shown potential for extending survival when used alongside surgery, particularly in gliomas, with some studies reporting a few months' increase in median survival. On the other hand, TTFields, especially when combined with chemotherapy, have shown a significant improvement in median overall survival in GBM patients, indicating a potential advantage over PDT in certain scenarios. CAP therapy is relatively new, with limited clinical data on survival rates. Preliminary studies suggest potential benefits in tumor control, but more comprehensive studies are needed to compare its effectiveness against established modalities like radiotherapy and PDT. Each physical therapy has its own unique advantages and limitations in terms of survival rates. Radiotherapy and TTFields have more robust data supporting their use in various brain tumor types, whereas ablation therapies and PDT are often more suitable for specific cases. CAP therapy, being in its nascent stage, requires further clinical validation to establish its comparative efficacy.

## 7 OTHER METHODS FOR BRAIN TUMOR TREATMENT

Physical therapy for brain tumors typically utilizes high‐energy radiation, temperature, light, electric fields, or other active substances to effectively disrupt and eradicate tumor cells. This treatment modality aims to precisely target the tumor area, thereby minimizing damage to surrounding healthy tissues. Other treatment modalities for brain tumors encompass surgical resection, medication therapies, and immunotherapy. Surgical resection entails the removal of tumor tissue to maximize tumor eradication. Medication therapies use drugs to actively impede the growth and spreading of tumor cells. Immunotherapy, on the other hand, focuses on modulating the immune system to combat tumor cell evasion, thereby augmenting the recognition and elimination of cancer cells.

### 7.1 Surgical resection

Surgical resection stands as the primary and most prevalent treatment for patients clinically diagnosed with brain tumors. The procedure aims to remove the tumor as much as possible while safeguarding the surrounding healthy brain tissue. The surgical excision of brain tumors serves to reduce mass effect, decrease tumor burden, improve response to other treatments, and provide tissue for diagnosis.^[^ ^181^ ^]^ Brain tumors can bring about pressure on surrounding brain tissue, leading to symptoms such as headaches, nausea, and seizures.^[^ ^182^ ^]^ Surgical excision can alleviate these symptoms by reducing the pressure caused by the mass effect by removing the tumor. Surgical removal of the tumor can reduce the amount of cancerous tissue in the brain, potentially halting the progression of the disease. It can also improve the efficacy of other treatments, such as radiation therapy, by reducing the tumor burden and making the remaining cancer cells more susceptible to these therapies.^[^ ^183^ ^]^ Ryken et al. suggested that repeating surgery was beneficial for patients with symptomatic local recurrence or progressive malignant gliomas. Patients diagnosed with GBM after a second surgery can expect their median survival time to fall within the range of 6 to 17 months.^[^ ^184^ ^]^ Moreover, surgical excision provides the necessary tissue for diagnosis, enabling healthcare professionals to determine the most suitable treatment course and to predict patient outcomes. The tissue can be examined under a microscope to verify the tumor type and its grade, aiding in the determination of treatment options and prognosis. The treatment of tumors is a complex and multifaceted process that depends on several critical factors, including tumor type, grade, location, size, age, and the overall health status of the patient.^[^ ^185^ ^]^ Although surgery is a feasible option for localized and early‐stage malignant tumors, adjuvant therapies such as radiation therapy, chemotherapy, and targeted therapy can help control tumor growth and achieve complete remission. The most effective course of treatment, however, depends on the tumor's type and molecular attributes. For instance, gliomas, metastatic brain tumors, meningiomas, and brainstem gliomas necessitate personalized approaches.

**FIGURE 21:**
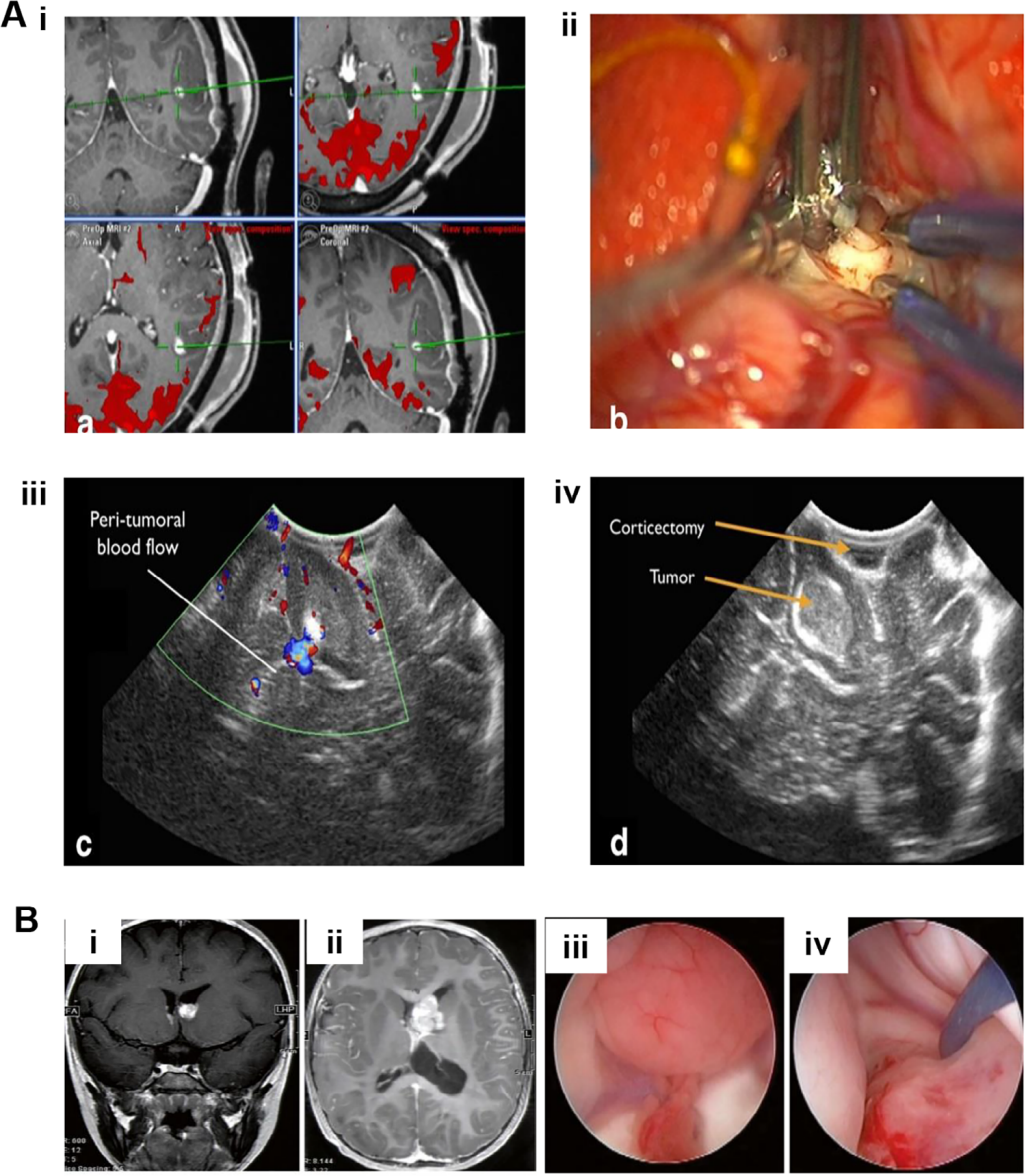
Surgical management for a brain tumor. (A) Craniotomy: A subcortical lesion in the left angular gyrus (i). Real‐time intraoperative ultrasound was used to precisely locate the lesion (ii) and surrounding blood vessels (iii). Minimal disruption to the surrounding cortex during lesion removal (iv). Reproduced with permission.^[^ ^188^ ^]^ Copyright 2015, Springer Nature. B) Neuroendoscopy: Coronal (i) and axial MRI showed a giant cell astrocytoma in the ventricle (ii). Endoscopic view of the lesion (iii). Tumor microsurgical resection assisted by endoscopy (iv). Reproduced with permission.^[^ ^189^ ^]^ Copyright 2017, Springer Nature.

Surgical resection is limited by the tumor's location and the risk of damaging critical brain areas. Innovations in surgical techniques and intraoperative imaging are required to enhance the safety and completeness of tumor removal. Craniotomy and neuroendoscopy are two surgical procedures used to treat brain tumors. Craniotomy, which involves the removal of a section of the patient's skull by a neurosurgeon, is the most prevalent method for brain tumor treatment. This surgical procedure is typically performed under general anesthesia to create a surgical window through which surgical intervention on the brain can be carried out.^[^ ^186^ ^]^ The primary objective of contemporary surgical interventions for brain cancer is to achieve maximal safe excision of the tumor while simultaneously preserving optimal neural function. Solheim et al. reported that 63% of the patients who underwent gross total resection (GTR) for GBM were deemed resectable.^[^ ^187^ ^]^ Moyiadi et al. evaluated the outcomes of 90 patients who underwent brain tumor resection surgery and achieved a final GTR rate of 88%. Furthermore, Almeida et al. introduced various techniques in neuro‐oncological surgery, inclusive of functional imaging, ultrasound surgery, intraoperative MRI, and intraoperative mapping techniques of cortical and subcortical regions. As illustrated in Figure 21A, intraoperative ultrasound technology provides real‐time information on tumor size, spatial positioning, and surrounding vasculature, enabling less invasive manipulation of the adjacent cortex during lesion removal.^[^ ^188^ ^]^

Neuroendoscopy, commonly referred to as “keyhole” neurosurgery, is a medical procedure used for partially or fully removing tumors from the fluid‐filled space in the brain's ventricles.^[^ ^189^ ^]^ An endoscope, comprising a long tube attached to a camera linked to a monitor and eyepiece, is used as a medical tool during the procedure. The endoscope used may be flexible or rigid. To gain access to the tumor, the surgeon creates a small hole in the patient's skull, known as a burr hole, and inserts the endoscope through it. The tumor is removed using the endoscope's tiny forceps and scissors, while the surgical team utilizes the endoscope and microsurgical tools to visualize the lesion, as demonstrated in Figure 21B.^[^ ^189^ ^]^ The endoscopic view enables real‐time visualization of the tumor and guides the microsurgical instruments to remove it. Postoperative imaging confirms the successful tumor removal. Neuroendoscopic biopsy therapy is reportedly highly effective, with a success rate of up to 96.0% for brain tumor treatment.^[^ ^190^ ^]^ In their analysis of 293 patients, Constantini et al. observed that 90.4% had pathological biopsy data. Moreover, only one patient died due to significant bleeding during surgery, resulting in a mortality rate of 17.9% for those undergoing surgery.^[^ ^191^ ^]^

Despite being widely used to treat brain tumors, surgery has several limitations. Patients may experience discomfort, bodily function loss, or cognitive impairment as a result of brain tissue removal around the tumor. Additionally, accurately determining tumor size and location before and during surgery is a significant challenge. Although various techniques, such as the use of microscopes, MRI, and three‐dimensional probe imaging, have been employed to assist surgeons in identifying and distinguishing between tumor cells and normal cells, complete removal of all tumor cells during the procedure is unlikely, thus leaving room for potential recurrence. This recurrence can be reduced by inserting carmustine chips locally in the resection cavity and undergoing postoperative radiotherapy or systemic chemotherapy.

### 7.2 Medication therapies

The treatment of brain tumors involves administering drugs that can effectively destroy tumor cells. These drugs can be delivered systemically, traveling through the bloodstream and reaching cancer cells throughout the body. Alternatively, they can be applied locally to a specific cancerous area or region of the body. The drugs employed for brain tumor treatment encompass chemotherapy and targeted therapy. An individual may receive either a single drug or a combination of multiple drugs simultaneously. Moreover, drug treatment may be part of a comprehensive plan, which could involve surgical procedures and/or radiation therapy. Chemotherapy's effectiveness is often limited by systemic side effects and its ability to penetrate the BBB. The development of drugs specifically designed for brain tumor pharmacokinetics is crucial to increase their efficacy and reduce toxicity.

#### 7.2.1 Chemotherapy

**FIGURE 22:**
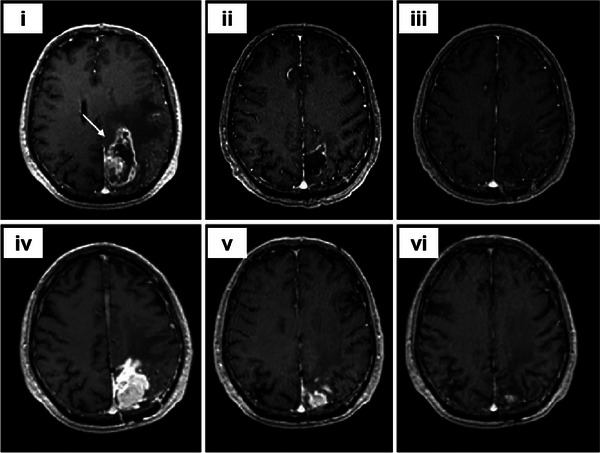
Axial T1‐weighted MRI scans were taken after fractionated stereotactic radiotherapy (FSRT) and temozolomide treatment for recurrent GBM: The arrow points to a large enhancing mass in the parietal lobe (i). Three weeks post‐surgery, the patient underwent radiotherapy and temozolomide treatment (ii). Post‐treatment imaging showed significant changes in the tumor (iii). After 20 months, the tumor recurred and was treated with FSRT and temozolomide (iv). Post‐treatment MRI scans at 2 months (v) and 6 months showed gradual tumor shrinkage (vi). Reproduced with permission.^[^ ^194^ ^]^ Copyright 2011, Springer Nature.

Chemotherapy is a cancer treatment that utilizes drugs to block the cell cycle of rapidly dividing cells, including cancer cells. This blockage occurs by interfering with DNA replication and cell division, ultimately leading to cell death.^[^ ^192^ ^]^ Chemotherapy is typically administered systemically, with drugs circulating throughout the body via the bloodstream and attacking cancer cells wherever they may exist. This treatment is commonly used for widely metastatic or highly malignant tumors. In the case of tumors that require local administration, such as those occurring in the brain, chemotherapy must be administered directly to the affected area through an artery, vein, muscle, skin, or orally to prevent drug diffusion throughout the body. A small needle is carefully inserted through the skin to access the Ommaya reservoir. Chemotherapy drugs are directly infused into the cerebrospinal fluid (CSF)‐filled space in the brain, enabling lower doses of chemotherapy to be administered by circumventing the BBB. The origins of chemotherapy trace back to the 1940s, when nitrogen mustard and folate antagonists were first used to treat tumors. Significant tumor regression was observed by Gilman at Yale University, opening the door to the era of chemotherapy.^[^ ^193^ ^]^ In the 1960s, chemotherapy was demonstrated as an effective adjuvant therapy when used alongside surgery and radiation therapy. It exhibited maximal anti‐tumor effects and minimal toxicity to normal tissues, consequently becoming the standard clinical treatment.^[^ ^11^ ^]^ After brain tumor surgery, chemotherapy is typically administered and sometimes combined with radiation therapy, as shown in Figure 22.^[^ ^194^ ^]^

The chemotherapy drugs most frequently used to treat brain tumors are temozolomide, carmustine, lomustine, procarbazine, and vincristine. Administrated in cycles, these drugs are followed by a period of rest to give the body time to recover. According to a report by the Korean Neurosurgical Society, it is recommended to administer temozolomide once a month for 6 to 12 months following radiation therapy for patients with GBM and high‐grade gliomas. This treatment has been shown to effectively prolong the survival period of GBM patients.^[^ ^195^ ^]^ The Gliadel wafer is a drug delivery method that places carmustine at the surgical site after tumor removal. The slow release of the drug has been demonstrated to improve tumor treatment effectiveness.^[^ ^196^ ^]^ Combining lomustine, procarbazine, and vincristine with radiation therapy has also been used to prolong the lives of patients with Grade III oligodendroglia tumors that have a common loss of 1*p*/19*q*.^[^ ^197^ ^]^ Chemotherapy has been shown to increase the lifespan of patients who have undergone radiation therapy, but who cannot have full tumor removal via surgery due to low‐grade tumors.^[^ ^198^ ^]^ Although chemotherapy can potentially be effective, it may not always serve as a reliable cure for cancer, especially when the tumor has progressed or metastasized to other parts of the body. Therefore, it is imperative for individuals diagnosed with brain tumors to have in‐depth discussions with their healthcare providers regarding the potential advantages and risks of undergoing chemotherapy.

The potential side effects of chemotherapy for brain tumors can be substantial and divergent, depending on factors such as the dosage, specific drug usage, and individual patient reactions. Such effects may include fatigue, an increased risk of infection, nausea, vomiting, hair loss, appetite loss, and diarrhea. Moreover, certain chemotherapy agents produce significant long‐term consequences, such as nerve or heart damage, which can hurt a patient's life quality. In many cases, these side effects will resolve after treatment. In rare instances, certain medications may lead to hearing loss or renal impairment, but the latter can potentially be alleviated by intravenously administrating additional fluids.

#### 7.2.2 Targeted therapy

Apart from conventional chemotherapy, physicians use targeted therapy as an alternative approach to treat cancer with medication, which specifically targets the genes, proteins, or tissue environment that support tumor growth and survival. This treatment approach aims to inhibit the proliferation and metastasis of tumor cells while minimizing adverse effects on healthy cells. Administering local anti‐cancer drugs to intracranial targets shows promise in achieving sustained concentrations, allowing for therapeutic effects.^[^ ^199^ ^]^ However, physical and physiological barriers within the BBB and blood‐cerebrospinal fluid barrier (BCSFB) hinder drug delivery to the central nervous system.^[^ ^200^ ^]^ To accurately and effectively deliver drugs to tumor locations, several traditional strategies have been employed,^[^ ^201^ ^]^ including the use of biochemical agents, ultrasound, and radiation to disrupt the central nervous system barrier. Additionally, invasive methods such as intrathecal injection, intraventricular injection, and intertumoral injection have been utilized. However, these methods are associated with severe neurotoxicity and neuropathological consequences. Given the limitations of traditional drug delivery strategies for brain tumors, interest in novel approaches has surged. A promising concept involves creating transient openings or disrupting BBB integrity by destroying tight connections. Liu et al. mention the use of a “two birds, one stone” strategy, where the nanomedicine is designed to penetrate the BBB and target GBM cells. It also highlights the ability of nanomedicine to enhance the immune response against GBM and increase the sensitivity of GBM tumor cells to TMZ.^[^ ^202^ ^]^ Although this approach exhibits great potential, its safety and efficacy require further evaluation.

**FIGURE 23:**
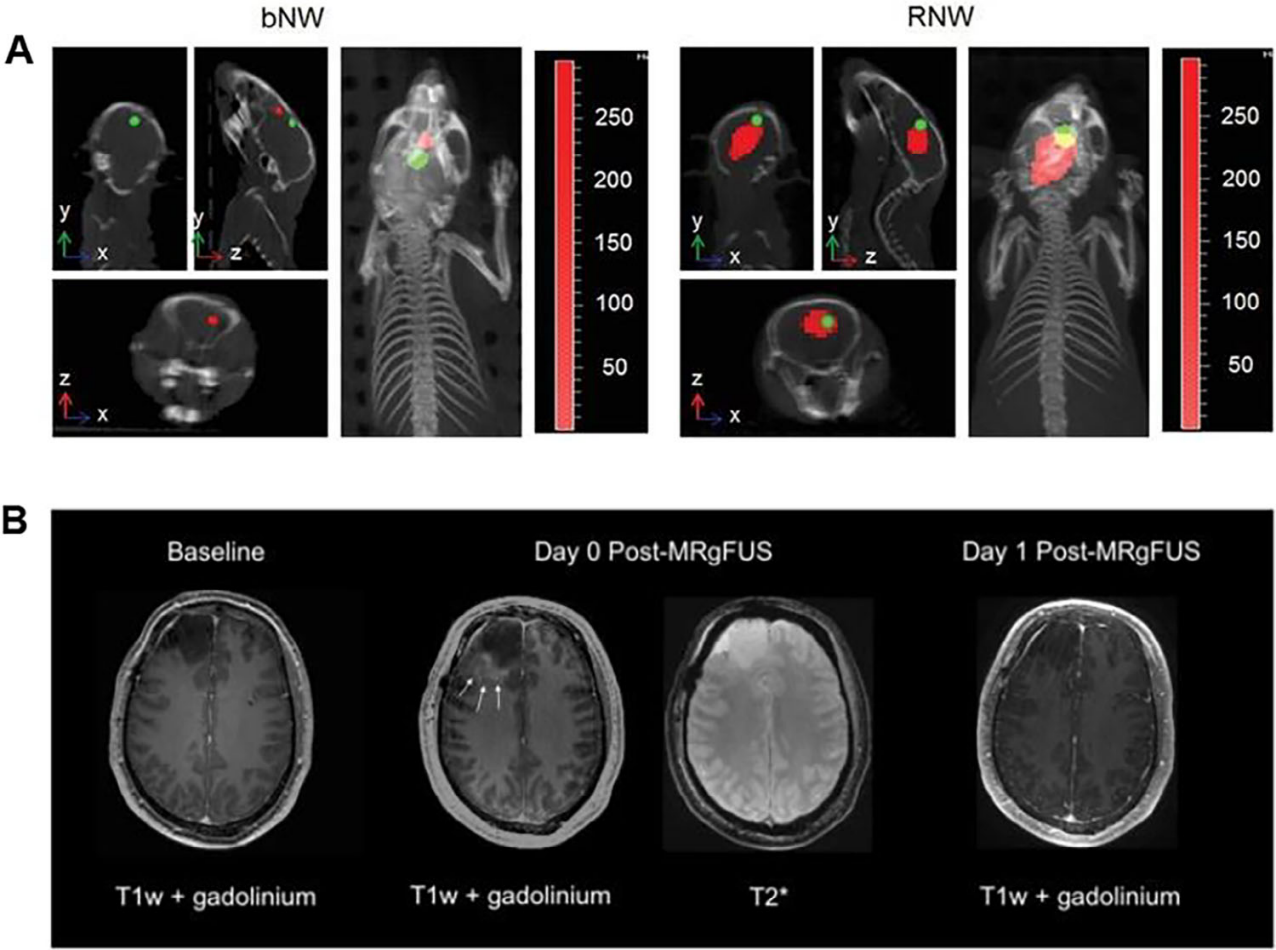
Targeted therapy for brain tumors. (A) In vivo distribution of micelles (RNW and bNW) and their specific targeting effects on tumor tissues in the U87‐Luc‐induced in situ brain tumor model. Reproduced with permission.^[^ ^206^ ^]^ Copyright 2016, Wiley‐VCH Verlag. (B) Ultrasound therapy delays tumor treatment progress in vivo. The images depict the patient in three different stages: 30 days before the ultrasound exposures (left), after the ultrasound exposures induced disruption of the BBB (middle), and 20 h after the BBB breach (right). Reproduced under the terms of the CC‐BY 4.0 license.^[^ ^211^ ^]^ Copyright 2019, The Authors, published by Springer Nature.

Nanocarriers are considered highly promising methods for targeted therapy of brain tumors.^[^ ^203^ ^]^ In contrast to conventional drug formulations, nanomedicine provides distinct benefits such as shielding against degradation, high drug solubility, high drug loading capacity, multifunctional surface modification, uniform size distribution, targeted drug delivery, and responsive drug release behavior.^[^ ^204^ ^]^ Several nanocarrier systems have been explored for brain tumor targeting, including polymer NPs, liposomes, dendrimers, nanomicelles, polymer vesicles, gold nanoparticles, nanogels, quantum dots, and magnetic NPs.^[^ ^205^ ^]^ As depicted in Figure 23A, a recent study has unveiled a novel, advanced worm‐shaped nanomicelle, mPEG‐b‐PDPA, which selectively responds to brain tumor micro‐environments, thereby stimulating degradation and facilitating drug release to target brain tumors.^[^ ^206^ ^]^ When compared to blank mPEG‐b‐PDPA micelles (bNW), mPEG‐b‐PDPA (RNW) micelles that are further modified with RGD‐DM1 exhibit a highly specific drug delivery capacity for specific brain tumors. The worm‐like structure of the micelles permits deeper penetration into 3D tumor tissues. Chakroun et al. conducted a review of recent improvements in nanocarrier‐based drug delivery systems (DDS) to address the key challenge of delivering a sufficient amount of therapeutic agents to the brain tumor site while minimizing potential side effects.^[^ ^185^ ^]^ The rapid progress of nanotechnology has facilitated the use of nanomedicine in biomedical applications, including enhancing drug delivery to the brain. Kim et al. provided insights into designing nanomedicines able to cross the BBB and deliver therapeutic agents to specific brain sites using various types of materials, including polymers, lipids, and inorganic compounds.^[^ ^207^ ^]^ The study summarized the latest challenges and prospects of nanotherapeutics for drug delivery to the brain.

Recently, magnetic resonance‐guided focused ultrasound (MRgFUS) has emerged as a promising technology for temporarily disrupting the BBB.^[^ ^208^ ^]^ Preclinical models have demonstrated that this approach can effectively open the BBB without causing any harm or permanent damage.^[^ ^209^ ^]^ According to Lipsman, this physical process of pulling apart tight junctions creates spaces between cells to allow circulating compounds in the blood to better cross into the brain.^[^ ^210^ ^]^ A recent study has reported that MRgFUS can temporarily open the BBB, as observed on T1‐weighted MRI in Figure 23B.^[^ ^211^ ^]^ The imaging revealed a reversible contrast agent enhancement in the brain parenchyma, indicating selective permeabilization of the BBB. Remarkably, the follow‐up imaging conducted one day later confirmed that the BBB had effectively closed, emphasizing the transient nature of the MRgFUS‐induced opening. These findings indicate that MRgFUS holds immense potential as a targeted and noninvasive approach for delivering therapeutics to the brain. Moreover, Todd et al. utilized focused ultrasound (FUS) to non‐invasively breach the BBB and effectively deliver chemotherapy drugs using microbubbles that mechanically vibrate to transport the drugs to specific areas.^[^ ^211^ ^]^ The combination of FUS and microbubbles enhances BBB permeability and improves drug transportation to the brain, resulting in reduced pathology and increased survival rates in preclinical disease models.^[^ ^212^ ^]^ Moura et al. demonstrated in a rat model that they can selectively and reversibly disrupt the BBB by opening tight junctions or inducing contraction of endothelial cells in a controlled manner, which can be reversed within 96 hours without causing damage to the surrounding central nervous system.^[^ ^213^ ^]^ Furthermore, Jones et al. developed an advanced three‐dimensional imaging system to monitor ultrasound emissions from oscillating microbubbles, a technique that temporarily opened the BBB without adverse effects.^[^ ^214^ ^]^ The study by Lao et al. highlights the innovative use of FUS to transiently open the blood‐brain barrier, enabling efficient delivery of CRISPR/Cas9 for targeted brain genome editing.^[^ ^215^ ^]^ This groundbreaking approach significantly enhances gene editing efficiency in the brain, offering promising potential for treating neurodegenerative diseases and advancing gene therapy applications in neuroscience.

### 7.3 Immunotherapy

**FIGURE 24:**
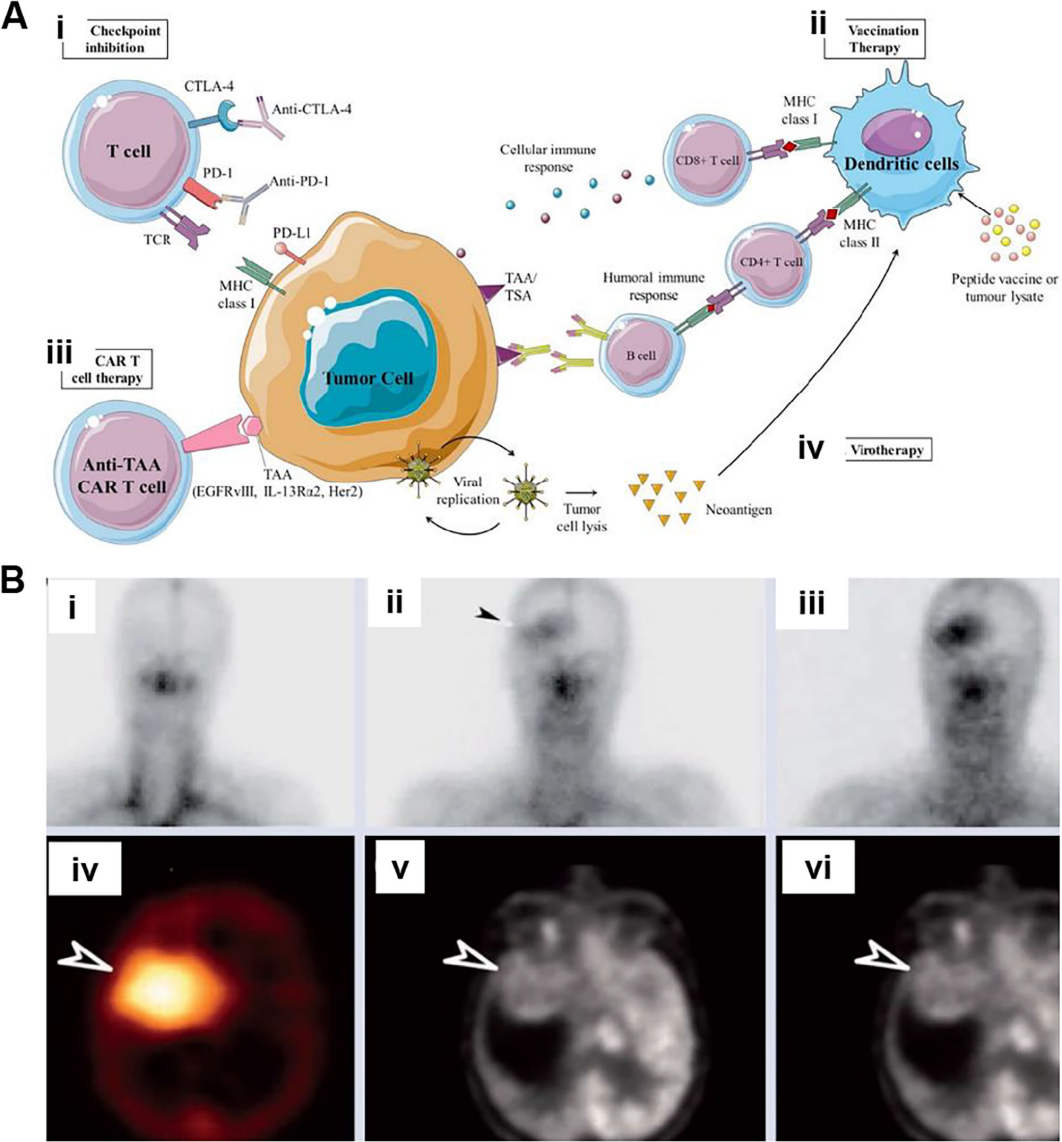
Immunotherapy for brain tumors. (A) Several types of immunotherapies: checkpoint inhibitors (i), vaccination therapy (ii), CAR‐T cell therapy (iii), and virotherapy (iv). Reproduced under the terms of the CC‐BY 4.0 license.^[^ ^221^ ^]^ Copyright 2021, The Authors, published by OAE Publishing Inc. (B) The enrichment of ch806 infusion in the head and neck of patients on day 0 (i), day 3 (ii), and day 7 (iii) is shown in the following imaging modalities. SPECT imaging (iv), 18F‐FDG positron emission tomography (v), and MRI of the brain (vi). Reproduced with permission.^[^ ^222^ ^]^ Copyright 2007, National Academy of Sciences, USA.

Tumor cells can evade immune system recognition through various mechanisms, including the secretion of immunosuppressive cytokines or the expression of inhibitory receptors, which curtail the immune response and prevent immune cells from recognizing and attacking the tumor cells.^[^ ^216^ ^]^ Immunotherapy works by modulating the immune system, enabling it to overcome these mechanisms and enhance cancer cell recognition and elimination. This approach involves natural or lab‐manufactured materials to improve, target, or restore immune system function, including checkpoint inhibitors,^[^ ^217^ ^]^ CAR‐T cell therapy,^[^ ^218^ ^]^ vaccination therapy,^[^ ^219^ ^]^ and virotherap^[^ ^220^ ^]^ (as illustrated in Figure 24A ^[^ ^221^ ^]^). T cells serve as a crucial line of defense in safeguarding the body against infections by targeting and eliminating cells recognized as “non‐self,” such as viruses and bacteria. T cells can also identify and destroy tumor cells that produce numerous mutated proteins, interpreted by T cells as “intruders.” Immunotherapy aims to activate anti‐tumor T‐cell responses, which have the potential to completely eradicate tumors and prevent their recurrence. Moreover, immunotherapy can counteract the immune‐suppressive effects exerted by tumor cells on the immune system. By utilizing checkpoint inhibitors such as anti‐PD‐1 or anti‐PD‐L1 antibodies, immunotherapy can effectively restore the activity of immune cells. Additionally, immunotherapy has the potential to activate memory cells in the immune system, enabling them to better recognize and attack tumor cells while also priming them for faster and more potent responses in the future. Notably, CAR‐T cell therapy genetically modifies a patient's T cells to express artificial receptors specific to certain antigens, enhancing the immune system's response against brain tumors.^[^ ^217^ ^]^

Targeted antibodies are a type of protein produced by the immune system that can be engineered to recognize specific markers on cancer cells. By binding to these markers, the antibodies can impede cancer activity, especially uncontrolled growth. Antibody‐drug conjugates (ADCs) carry anti‐cancer drugs that are specifically delivered to tumors. Bispecific T cell‐engaging antibodies (BiTEs) are capable of binding to both cancer cells and T cells, effectively enhancing the response of the immune system. According to Figure 24B, the clinical study of the ch806 antibody, which specifically targets the epidermal growth factor receptor variant III (EGFRvIII) antigen found exclusively on the surface of GBM, demonstrated increased BBB permeability after systemic administration.^[^ ^222^ ^]^ Fecci et al. have pointed out that immunotherapy may be a viable option for addressing moderate immunogenicity and the desire for treatment specificity in GBM while staying within the normal brain range of toxicity aversion in the brain.^[^ ^223^ ^]^ Further studies are needed to ascertain the effectiveness and safety of these immunotherapy strategies.

Overall, immunotherapy has shown promising outcomes in treating brain tumors. For example, checkpoint inhibitors have demonstrated improved survival rates in patients with recurrent glioblastoma. Targeted antibodies have also yielded promising results in early clinical trials, with some patients experiencing complete tumor remission. Further research is required to gain a full understanding of the long‐term effects of immunotherapy on brain tumors. The brain's unique immune privilege poses a major challenge for effective immunotherapy. Overcoming the immunosuppressive tumor microenvironment and enhancing the delivery of immunotherapeutic agents are key areas for future research. Recent studies have focused on developing PSs with non‐linear optical properties, enabling activation by near‐infrared light for deeper tissue penetration.

### 7.4 Cutting‐edge research

The clustered regularly interspaced short palindromic repeat (CRISPR) technology has marked a revolutionary step in cancer research, offering a new frontier in brain tumor therapy. CRISPR, a gene‐editing tool, allows for precise alterations of the genome, providing a potential pathway to correct genetic mutations that contribute to the development and progression of brain tumors. Recent studies have demonstrated the capability of CRISPR to target and modify specific genes associated with brain cancer, paving the way for more effective and personalized treatment strategies.^[^ ^224^ ^]^ The mechanisms of action of CRISPR in brain cancer involve targeting specific genes linked to oncogenesis. By editing these genes, researchers can potentially deactivate or knockout oncogenes or repair malfunctioning tumor suppressor genes, thereby inhibiting tumor growth.^[^ ^225^ ^]^ Studies have shown promising results in using CRISPR to disrupt key pathways in glioblastoma cells, indicating a potential for more effective treatments. Recent developments in CRISPR technology have focused on enhancing delivery mechanisms to target tumors more effectively. Innovations such as nanoparticle‐based delivery systems and viral vectors are being investigated to improve the precision and efficiency of CRISPR gene editing.^[^ ^226^ ^]^ Furthermore, combining CRISPR with other therapeutic modalities, such as chemotherapy or immunotherapy, is a promising approach to synergistically enhance treatment efficacy.^[^ ^226b^ ^]^ These combinations could lead to more effective strategies for combating brain tumors and improving patient outcomes. This precision targeting is crucial in brain cancer, where genomic mutations play a significant role in tumor behavior. However, one potential side effect is off‐target gene editing, where unintended genetic alterations may occur, potentially leading to unforeseen complications. Ensuring specificity and minimizing these off‐target effects are critical areas of ongoing research.

Recent advancements in molecular biology have led to the identification of new molecular targets for brain cancer treatment. One notable development is the use of nanoparticle‐based treatment that targets multiple culprits in GBM, such as oncomiRs miR‐10b and miR‐21.^[^ ^227^ ^]^ This approach has shown promise in sensitizing cancer cells to chemotherapy and radiotherapy, potentially leading to more effective treatment outcomes. Such advancements in molecular targeting hold great potential for enhancing the efficacy of brain cancer treatment and improving patient outcomes.^[^ ^228^ ^]^ In addition to targeting specific genetic mutations, recent research has focused on novel therapeutic approaches such as oncolytic viruses, CAR T‐cell therapy, and the use of NPs for drug delivery. Oncolytic viruses are engineered to selectively infect and kill cancer cells, while sparing normal cells.^[^ ^229^ ^]^ CAR T‐cell therapy involves genetically modifying patients' T‐cells to recognize and attack tumor cells.^[^ ^230^ ^]^ NPs are being explored for their ability to cross the BBB and deliver therapeutic agents directly to the tumor site.^[^ ^231^ ^]^ The concept of personalized medicine has gained significant traction in brain tumor treatment. By analyzing individual tumor genetics, researchers are developing personalized treatment plans that target specific molecular profiles of each patient's tumor. This approach aims to improve treatment efficacy and reduce side effects by tailoring therapy to each patient's unique tumor characteristics. Advanced imaging techniques and artificial intelligence (AI) are increasingly being integrated into brain tumor treatment planning.^[^ ^232^ ^]^ AI algorithms can analyze imaging data to identify tumor characteristics, predict treatment responses, and assist in surgical planning. These technologies enhance the precision of treatment and offer the potential for better outcomes.

## 8 CONCLUSIONS AND CHALLENGES

The complexity of the brain structure and function, as well as the tumor location and type, pose significant challenges to treating brain tumors. The brain is a delicate organ that regulates vital bodily functions, and the tumors within it can cause extensive damage to critical neural or vascular structures, presenting significant challenges for surgeons. Furthermore, the BBB limits the access of many drugs and therapies to the brain, creating obstacles to delivering effective treatments. Therefore, treating brain tumors is a complicated and demanding process that requires expertise in the field and advanced technology. The treatment of brain tumors depends on several factors, including tumor type, location, size, and grade, and also considers age, general health status, and personal preferences. This review has covered treatment options for brain tumors, including physical therapies and other advanced treatment methods. Surgical resection entails removing tumor tissue through surgical procedures and is the preferred approach for addressing extensive, benign, or minimally invasive tumors, as well as cases necessitating immediate decompression. Surgical treatment eradicates the tumor, allowing for pathological examination and analysis to identify the tumor type and monitor disease progression. Although surgical resection can effectively treat brain tumors, it may also result in detrimental effects on the surrounding healthy tissue, leading to neurological deficits and cognitive impairments. Moreover, some tumors may be challenging to remove completely due to their location in the brain.

Radiation therapy has seen advancements in recent years, including the use of advanced techniques such as SRS and IMRT. These techniques allow for precise targeting of tumor tissue while minimizing damage to surrounding healthy tissues. Another advancement is proton therapy, which uses protons instead of traditional X‐rays for radiation therapy. Proton therapy provides more precise targeting and delivers higher radiation doses, increasing the effectiveness of tumor cell destruction. Moreover, brachytherapy offers precise targeting, reduced side effects, shorter treatment duration, increased treatment effectiveness, and improved QOL in the treatment of cancer. However, radiation therapy does have its limitations. It may cause side effects such as fatigue, hair loss, skin irritation, and damage to normal brain tissue, potentially leading to cognitive impairments. Furthermore, not all tumors respond well to radiation, especially those located in critical or inaccessible areas of the brain. Lastly, multiple radiation treatments over a prolonged period may be necessary, which can be inconvenient for patients. Other treatments, such as microwave, LITT, cryosurgery, and photodynamic therapy, use heat or cold to destroy cancer cells. These treatments can be effective in some cases, but they can also cause damage to the healthy tissue surrounding the tumor and may not be suitable for all types of brain tumors. Tumor‐treating fields use external electrical fields to disrupt cancer cell division. While it has shown some promise in clinical trials, its utilization can be burdensome for patients and lead to skin irritation or discomfort. CAP jets leverage ionized gas to damage and destroy cancer cells. Even though preclinical studies have been encouraging, they are still undergoing early‐stage development and require further testing.

Chemotherapy has some therapeutic benefits for faster‐growing brain tumors, such as medulloblastoma and lymphoma, but proves to be of little effectiveness for highly aggressive tumors like glioblastoma. Additionally, it can cause systemic toxicity and may not cross the BBB effectively, further diminishing its efficacy. Targeted therapy is a drug treatment that specifically targets tumor cells, using its specific mechanism of action to impede the growth and division of tumor cells. Common targeted therapy drugs comprise tyrosine kinase inhibitors and anti‐angiogenic agents, which are administered via intravenous injection or taken orally to aim for specific, abnormal signaling pathways or targets for treatment. Research has demonstrated that targeted therapy is efficacious and safe. Combination therapies, which combine targeted agents with other treatment modalities, have also shown promise in improving treatment outcomes and overcoming resistance to targeted therapy. Additionally, targeted therapy may only be effective in tumors with specific genetic alterations, which means it may have limited applicability to tumors lacking these targets. Alternatively, immunotherapy is a treatment modality that utilizes the immune system to attack tumor cells, which includes but is not limited to cellular immunotherapy, protein therapy, and gene therapy. One of the promising advancements is the use of chimeric antigen receptor (CAR) T‐cell therapy. This therapy involves genetically modifying immune cells to specifically target tumor cells, resulting in promising results. Additionally, checkpoint inhibitors have shown efficacy in enhancing the immune system response against brain tumors. However, it has yet to validate its effectiveness for brain tumors, despite yielding promising results in other cancer types.

In conclusion, each of the current brain tumor treatments has its own specific limitations, including restricted efficacy, risk of damage to healthy brain tissue, treatment resistance, and/or systemic toxicity. Therefore, the development of more effective, targeted, and/or combined therapies might be beneficial to patients with brain tumors.

## CONFLICT OF INTEREST STATEMENT

The authors declare no conflicts of interest.
